# Bioprospecting and Challenges of Plant Microbiome Research for Sustainable Agriculture, a Review on Soybean Endophytic Bacteria

**DOI:** 10.1007/s00248-022-02136-z

**Published:** 2022-11-01

**Authors:** Modupe Stella Ayilara, Bartholomew Saanu Adeleke, Olubukola Oluranti Babalola

**Affiliations:** 1 Food Security and Safety Focus Area, Faculty of Natural and Agricultural Sciences, North-West University, Private Bag X2046, Mmabatho, 2735 South Africa; 2 Department of Biological Sciences, Microbiology Unit, Faculty of Science, Olusegun Agagu University of Science and Technology, PMB 353, Okitipupa, Nigeria

**Keywords:** Endosphere, Food security, Leguminous crop, Nitrogen-fixing bacteria, Plant growth promotion, Soybean microbiome

## Abstract

This review evaluates oilseed crop soybean endophytic bacteria, their prospects, and challenges for sustainable agriculture. Soybean is one of the most important oilseed crops with about 20–25% protein content and 20% edible oil production. The ability of soybean root-associated microbes to restore soil nutrients enhances crop yield. Naturally, the soybean root endosphere harbors root nodule bacteria, and endophytic bacteria, which help increase the nitrogen pool and reclamation of another nutrient loss in the soil for plant nutrition. Endophytic bacteria can sustain plant growth and health by exhibiting antibiosis against phytopathogens, production of enzymes, phytohormone biosynthesis, organic acids, and secondary metabolite secretions. Considerable effort in the agricultural industry is focused on multifunctional concepts and bioprospecting on the use of bioinput from endophytic microbes to ensure a stable ecosystem. Bioprospecting in the case of this review is a systemic overview of the biorational approach to harness beneficial plant-associated microbes to ensure food security in the future. Progress in this endeavor is limited by available techniques. The use of molecular techniques in unraveling the functions of soybean endophytic bacteria can explore their use in integrated organic farming. Our review brings to light the endophytic microbial dynamics of soybeans and current status of plant microbiome research for sustainable agriculture.

## Introduction 

Globally, diverse oilseed crops are cultivated for edible oil production to safeguard humans from malnutrition and related illnesses [1]. Their production rate differs from one country to another due to adaptation and growth under different weather conditions by region (e.g., temperate, tropical, and subtropical) [2]. The major type of oilseed crops are canola, groundnut, palm oil, sunflower, soybean, peanut, rapeseed, and cottonseed [3]. In 2020/2021, statistics of USDA showed an account of 362.05 soybeans, 68.87 rapeseed, 49.46 sunflower seed, 47.79 peanuts, and 41.80 of cottonseed, 19.96 palm kernel, and 5.75 copra world oilseed production (million metric tons) with soybean estimated of about a 90% production in the USA [4]. Also, in Sub-Saharan Africa, Nigeria produces and exports a larger percentage of soybean annually.

Soybeans are leguminous plants in the family Fabaceae. Interest in soybean cultivation relies on their economic value, the edible oil-producing potential of about 20%, and protein content of 20–25% [5]. Notably, soybeans serve as an inexpensive and excellent source of high-quality edible oil and protein for humans as compared to other leguminous crops and animal protein [6], and can be a supplement food source for livestock. Yet, soybean’s market value and maximum utilization are less explored in many countries [7]. Soybean can be processed into composite food products, substituting animal proteins, i.e., eggs, meat, and milk.

The uncertainties and challenges facing soybean cultivation may include poor and inefficient farming systems, drought, disease invasion, pest attack, lack of disease-resistant cultivars, etc. [8–10]. Diseases such as stem and root blight, bacterial leaf blight, downy mildew, bacterial pustule, rust, purple seed stain, frog-eye leaf spot, brown spot, charcoal rot, and soybean mosaic virus are the most common peculiar to soybean [11]. The control of disease in plants and crops under storage can be achieved by either biological, chemical, or physical means. Therefore, adopting proper control measures against phytopathogens in soybean can sustain plant health and crop productivity.

From antiquity, farmers adopted diverse cropping systems (crop rotation, mixed farming, organic farming, etc.) and agricultural practices (e.g., agrochemicals, irrigation, and harrowing) to mitigate bottlenecks limiting the cultivation of soybean and other food crops. Over time, agrochemical use has been a major concern to environmentalists, ecologists, and microbiologists due to the negative impact on the ecosystem [12, 13]. The peculiarity of these challenges is not limited to soybean cultivation alone, but other economical food crops.

In recent times, research efforts are on the increase to devise a sustainable means of improving soybean, and other food crop production in order to help solve food scarcity, hunger, and malnutrition [14]. Because of the environmental threats posed by the synthetic fertilizer application and the incessant population increase, the need to employ biorational approaches and sustainable measures to enhance soybean production has become imperative. Naturally, soybean houses endophytic microbes capable of increasing the nitrogen pool in the soil to enhance plant nutrition for higher productivity [15]. The natural occurrence of these nitrogen-fixing bacteria is a promising way to reclaim lost soil nutrients for food production to meet the demand of the ever-growing population and relieve farmers of the cost and over-dependence on chemical fertilizers by farmers. Thus, harnessing endophytic bacteria as bioinoculants to oppose chemical fertilizers is critical as the best alternative.

The plant root endosphere represents discreet regions occupied by diverse, endophytic microorganisms [16], where these microbes exhibit mutualistic, neutral, or antagonistic relationships with the host plants. The emphasis on the root-associated bacteria will be most considered in this review, as soybean root nodules naturally contain diverse nitrogen-fixing bacteria (NFB) [17]. The complementary effects of root-associated bacteria and root nodule NFB can positively influence plant growth and survival under nitrogen-limiting soils [18]. Here we emphasize that the nitrogen-fixing potential of endophytic bacteria in leaves, stems, seeds, flowers, ovules, etc. may be of greater importance in plant growth when compared to root nodules NFB only. Nevertheless, comparative studies of these bacteria from various plant organs upon inoculation under greenhouse and field experiments are required to ascertain this claim, requiring further studies.

The molecular insights into plant–microbe interactions have unveiled important functions of some endophytic microbes, which suggests their maximum exploration as bioinoculants in sustaining plant growth and health [19]. For instance, a few beneficial nodule endophytic microbes associated with soybeans have been assessed under greenhouse and field trials to enhance soybean yield and in vitro screening for their antimicrobial properties against phytopathogens [20].

The interdependence of endophytic bacteria with the host plants confers beneficial effects in soybeans and other food crops, such that it stimulates plant growth promoters, antibiosis activity against phytopathogens for plant health, defense against oxidative stress, and yield enhancement without any pathogenic effects [21, 22]. Limited information is available in the literature on the plant growth stimulation and biocontrol potential of endophytic bacteria inhabiting soybean, thus limiting their ecological services. Nevertheless, exploring endophytic bacteria as bioinoculants can provide several opportunities in mitigating diverse agricultural problems, such as biotic and abiotic stress, and climate change. Furthermore, addressing the challenges and uncertainties limiting plant microbiome biotechnologically will ultimately reveal the amazing realities of incorporating endophytic resources from soybean and other food crops into agricultural management. Our review brings to light the endophytic microbial dynamics of soybeans and current status of plant microbiome research for sustainable agriculture.

## The Soybean Microbiome

It is essential to evaluate the diversity and population of endophytes in soybean plants in different environments, as a knowledge of this would serve as a background to promote their usage as biofertilizers, soil amendments, plant growth enhancers, and biocontrol agents with the overall aim of increasing different plant yield. Despite the success recorded in soybean’s endophytic microbiome research with promises to achieve future agricultural productivity [10, 15, 23], there is still a need for further studies. Sustaining plant health is paramount, as it is relatively mirrored in crop yield. The antimicrobial compounds and metabolites naturally found in economical plants, coupled with the biocontrol potential of some endophytic microbes, can contribute to plant health by reducing plant pathogenicity [24]. Inefficient control of plant pathogens results in yield loss to crops [25]. To help ameliorate these threats, in vitro screening of novel endophytic bacteria from economic plants for antimicrobial activity became important in identifying targeted biocontrol agents to specific pathogens in the host plants [26].

Taking account of key certain environmental factors that influence microbial community structure by monitoring different ecological niches is vital to ascertain specific environmental factors influencing microbial diversity in plants. For instance, in the phyllosphere, a limited supply of nutrients, ultraviolet light, humidity, temperature, oxygen concentration, pH, etc. influence the microbiome in this niche [27]. In the root endosphere, pathogens, nutrient deposition, and versatility might be key factors influencing the diversity of the microbes in different plants. The effect of ultraviolet “B” has been reported to influence the bacterial community structure in the soybean phyllosphere [28]. The factors (geographical location, carbohydrates, amino acids, and other soil nutrients) influence the microbial diversity in the root endosphere [29]. The soil-inhabiting microbes and some phyllosphere endophytic microbes can withstand high ultraviolet radiation due to the presence of pigments, i.e., melanin, xanthomonadine, and carotenoids [30]. The microorganisms found in the same ecological niche can be differentiated based on their characterization, genetic composition, and metabolic activities [30].

Plant endosphere ecology comprises microbial domains found in the below (root, sometimes seeds) and above (stem, leaf, seed, flower, and ovule) plant parts [31]. The microbial population and diversity in the plant root may be dissimilar compared to the other plant parts. The root endophytes are influenced by the exudate-secondary metabolites released into the soil-root environment [32]. Mina, Pereira, Lino-Neto, and Baptista [33] stated that the endophyte diversity in the different organs of a particular plant is mediated by the physical and chemical properties. This claim was relative to soybean as de Almeida Lopes, Carpentieri‐Pipolo, Oro, Stefani Pagliosa, and Degrassi [34] observed a similarity in the diversity of microorganisms in soybean.

### Endophytic Bacteria Associated with Soybeans

Studies on the functional traits exhibited by endophytic bacteria associated with soybean and *Arabidopsis* aim to reveal their significance in agriculture, industry, and medicine [15, 35]. The effects of some endophytic bacteria from legumes and other food crops on plant growth are presented in Table 1. Hence, the advantages of beneficial endophytic bacteria (e.g., plant production, growth, secondary metabolites) found in different food crops from various plant habitats remains crucial in plant growth promotion, inducing plant tolerance to harsh environmental conditions and disease control. These researchers reported *Citrobacter freundii* and *Enterobacter asburiae* from the root and stem; *Kosakonia cowanii*, *Pantoea agglomerans*, and *Variovorax paradoxus* from the root and leaf; *Staphylococcus aureus* from the stem and leaf; and *Enterobacter ludwigii* from the root, stem, and leaf of soybean. Likewise, Dubey, Saiyam, Kumar, Hashem, Abd_Allah and Khan [15] and Brunda, Jahagirdar, and Kambrekar [36] also isolated *Bacillus pumilus* from the stem and leaf of the soybean plant, which aligns closely with the claims of de Almeida Lopes, Carpentieri‐Pipolo, Oro, Stefani Pagliosa, and Degrassi [34], who observed similar bacterium in different organs of soybean. Hence, it is crucial to carry out more research to have a deeper understanding of the inherent factors affecting the diversity of endophytes in different plant parts for maximum exploration in solving agricultural problems.

**Table 1:** Effects of some endophytic bacteria from legumes and other food crops on plant growth

Plants	Endophytic bacteria	Effect on plants	References
Rapeseed	*Bacillus megaterium*, *B. pumilus*, *B. safensis*	Increased plant weight and shoot length compared to un-inoculated, fungal pathogen suppressiveness	[161, 162]
	*Pseudomonas*, *Paenibacillus*	Enhance shoot wet and dry weight	[163]
	*Pseudomonas putida*, *P. brassicacearum*	Improvement of crop yield under salt stress, plant growth promotion	[164, 165]
Soybean	*Alcaligenes faecalis*, *Paraburkholderia megapolitana*, *Stenotrophomonas maltophilia*	Bioremediation, plant growth promotion under drought stress	[166]
	*Bacillus cereus*, *Pseudomonas otitidis*, *Bradyrhizobium japonicum*, *Stenotrophomonas rhizophila*	Salt stress reduction, plant growth stimulation, yield enhancement	[15, 167, 168]
	*Bacillus amyloliquefaciens*	Osmolyte synthesis for plant growth under salt stress, crop yield improvement	[23, 169]
Groundnut	*Pseudomonas*	Biostimulation of plant growth and biocontrol for plant diseases	[170]
Canola	*Paenibacillus*, *Peribacillus*	Nitrogen fixation, plant growth promotion	[163, 171]
	*Micrococcus yunnanensis*, *Stenotrophomonas chelatiphaga*	Enhanced plant yield index (weight, root, and shoot), plant growth stimulation	[172]
Peanut	*Paenibacillus glycanilyticus*, *Pantoea dispersa*	Biofilm production, enhanced amylolytic capability, biomass increase in root nodules and jasmonic acid content, enhanced nodule formation, and growth	[173]
	*Bacillus velezensis*, *B. siamensis*, *B. subtilis*, *B. tequilensis*, *Rhizobium mayense*, *Pantoea*, *dispersa, Kosakonia oryzae*	Nodule formation, secondary metabolite biosynthesis, biocontrol activity, enhance crop yield	[174, 175]
	*Chryseobacterium indologenes*, *Pseudomonas aeruginosa*, *Enterobacter ludwigii*, *E. cloacae*, *Klebsiella variicola*, *K. pneumoniae*	Nitrogen fixation, plant growth promotion	[118]
	*Bacillus amyloliquefaciens*	Biocontrol potential	[176]
	*Enterobacter* spp., *Serratia* spp.	Phosphate mineralization, biostimulation of plant growth	[177]
Sunflower	*Bacillus cereus*, *Stenotrophomonas indicatrix*	Improve crop yield	[40, 90]
	*Exiguobacterium auranticum*, *Paenibacillus* spp.	Plant growth promotion and biocontrol	[21]
	*Pseudomonas lurida*	Phytoremediation	[178]
	*Acinetobacter bouvetii*	Phytostimulation, bioremediation, induced tolerance to chromate stress	[179]
Cottonseed	*Rhizoctonia solani*	Secretion of secondary metabolites, biocontrol activity, induced systemic resistance	[180]
	*Paenibacillus xylanilyticus*, *Paenibacillus polymyxa*, *Bacillus subtilis*	Biocontrol against soil-borne pathogens	[181]
	*Rhodococcus erythropolis*, *Rhizobium* spp., *Burkholderia*, *Sphingomonas* spp.	Phytodegradation, which increases the soil organic matter and soil nutrient	[182, 183]
	*Bacillus subtilis*, *B. velezensis*, *B. amyloliquefaciens*	Plant growth promotion	[184]
Maize	*Pseudomonas brenneri*, *Ewingella americana*, *Pantoea agglomerans*	Biocontrol activity against *Fusarium graminerum*. Plant growth improvement and yield enhancement	[185]
	*Lysinibacillus* spp., *Paenibacillus dendritiformis*, *Burkholderia anthina*, *Pseudomonas aureginosa*, *Bacillus* spp*.*, *B. subtilis*, *B. velensis*, *Staphylococcus arlettae*	Protection of maize seedlings against pathogenic fungus, *Fusarium verticillioide* by expression of defensive genes. Production of growth traits which enhanced plant growth	[186]
	*Burkholderia phytofirmans*, *Enterobacter* sp.	Enhanced the growth and physiological status of maize seedlings, reduced the effects of drought stress on maize and their photosynthesis rate	[187]
Rice	*Curtobacterium oceanosed-imentum*, *Curtobacterium luteum*, *Enterobacter ludwigii*, *Bacillus cereus*, *Micrococcus yunnanensis*, *Enterobacter tabaci*	Alleviate the effect of salt stress by the expression of salt stress genes and enhanced rice yield due to phytohormone production	[188]
	*Pantoea ananatis*	Contribute to plant growth, enhanced rice chlorophyll, total soluble protein, and proline contents, and improve the salt tolerance of rice seedlings	[189]
	*Bacillus oryzicola*	Biocontrol activity against the bakanae that causes seed-borne disease of rice by exhibiting induction of systemic resistance against the pathogen through primed induction of the jasmonic acid pathway	[190]
Wheat	*Azotobacter chroococcum*, *Acinetobacter guillouiae*	Enhanced yield biomass, iron and zinc content, and plant growth enhancer	[123]
	*Bacillus megaterium*, *B. subtilis*	Biocontrol activity against *Fusarium graminearum* causing *Fusarium* head blight and mycotoxin, deoxynivalenol	[191]
	*Burkholderia gladioli*, *Bacillus aryabhattai*, *B. altitudinis*, *B. wiedmannii*, *Pseudomonas aeruginosa*	Enhanced plant growth, reduce drought and salinity stress, and biocontrol activity against pathogens	[192]
Sorghum	*Ochrobactrum* spp., *Microbacterium* spp., *Enterobacter* spp., *Enterobacter* cloacae	Enhanced plant growth and induced stress tolerance	[193]
Pea	*Stenotrophomonas maltophila*, *Pseudomonas aeruginosa*, *Bacillus subtilis*	Biocontrol activity against phytopathogenic fungus, *Rhizoctonia solani* causing damping-off disease in cotton seedlings	[180]
	*Achromobacter xylosoxidans*, *Bacillus thuringiensis, B. cereus*, *B. subtilis*	Improve plant growth, yield biomass, biocontrol *Fusarium solani* causing plant root rot and boosting plant tolerance to salt stress	[194]

The selection of endophytic bacteria based on taxonomy and functions can help understand diverse bacteria communities in different plants [37]. Plants of the same species may have different bacteria compositions and associations, depending on the location, genotype, cropping system, climatic conditions, and growth stage [38].

The genomic data available on the microbes from soybean with unique metabolic features reveal their genetic variation. The notable genes involved in flagella biosynthesis (*flg*, *fil*, *flh*), chemotaxis (*che*ABRVWZ, *mpc*), IAA synthesis (*trp*ABCDE), nitrogen fixation (*isc*U), and phosphate solubilization (*pst*ABCS) identified in the genome of *Pseudomonas fluorescens* BRZ63 isolated from rapeseed may be responsible for the bacterium functions in enhancing plant growth and disease control [39]. A study by Adeleke, Ayangbenro, and Babalola [40] reported genes involved in nitrogen fixation, phosphate transport and solubilization, siderophore production, secretion systems, iron transport, flagella, flagella biosynthesis, and phytohormones in the genome of endophytic *Bacillus cereus* T4S isolated from sunflower, which enhanced sunflower yield. Furthermore, studies should also be intensified on soybean to unravel the genes in their different endophytes, enhancing plant growth.

Plant–microbe cooperation can modulate the transfer of certain genetic traits in the host plant by genome modulation, which may assist plants in acquiring novel traits and in boosting their adaptation mode of actions in diverse environments. The level of genetic communication in the root-soil interface facilitates microbial infiltration into the plants [41]. However, the similar genetic complexity between rhizosphere microbes and endophytic microbes provide new insights into their colonization pattern into the root endosphere and become endophytes [42]. Therefore, the mechanisms employed by soybean endophytic microbes in plant growth promotion need to be understood to ascertain their roles in the plant endosphere.

### Endophytic Fungi Associated with Soybean

Providing information on endophytic fungi (EF) inhabiting the root of soybean can help unravel the prospects of soybean in sustainable crop production. The plant growth–promoting attributes of bacteria and fungi inhabiting the root of plants may share significant similarities depending on the sample type, isolation source, and growth conditions [43, 44]. EF employs multifunctional strategies for plant growth and protection against biotic and abiotic stressors [45]. Unraveling the community structure and complex plant–microbe synergies in the host plants has made the science of endophyte interesting as a way of maximizing their bio-products (bioinoculants) to ensure food security [46, 47].

The EF which forms part of plant lifestyle with a strong affinity in the root endosphere due to the presence of cell organelle (mycelia) can be explored in agriculture [48–50]. Despite the ecological services of plant-associated EF, there is still a need to further investigate EF associated with soybean. For instance, the biocontrol potential of EF isolated from rapeseed against *Botrytis cinerea* and *Sclerotinia sclerotiorum*, which causes gray mold and *Sclerotinia* stem root, has necessitated further their exploration [51]. A study by Sallam, Ali, Seleim, and Bagy [10] reported antagonistic activity of the endophytic fungus *Trichoderma* spp. isolated from the soybean against *Rhizoctonia solani*, which reduces their effect on soybean yield under greenhouse experiments. Other research findings (to mention but a few) on the plant growth promotion and antifungal attributes of EF against plant pathogens were evident in literature due to phytohormone and metabolite secretions [25, 51–53].

The biotechnological potential of diverse EF in the production of therapeutic agents and antibiotics revealed their beneficial effect on plant immunity and growth enhancement [54]. The mechanism of action and factors influencing the diversity of root-associated endophytic bacteria and root-associated EF may be similar, possibly based on the same source of identification. Some identifiable EF isolated from the root, stem, and leaves of soybean with detailed biological activities for sustainable plant health includes *Trichoderma asperellum*, *T. longibrachiatum*, and *T. atroviride* [10], *Colletotrichum* spp., *Pestalotiopsis* spp., *Botryosphaeria* spp., *Diaporthe* spp. [55], and *Fusarium*, *Alternata* [56]. Despite their multifaceted attributes in plant growth promotion, disease suppressiveness, stress alleviation, metal reduction, and nutrient mineralization [57–59], there is still a need for more studies into the EF colonizing the root of soybean.

## Methodologies and Bottlenecks Limiting the Endophytic Study

The identification of endophytes in their host plants is somewhat difficult because some endophytic microbes might not be easy to culture in the laboratory [60], while some are viable but non-culturable. Hence, the use of culture-dependent and culture-independent methods remain important as the case may be. In the use of culture-dependent methods, the population of microbes are easily evaluated, while in contrast, the culture-independent methods are more useful in assessing the entire microbiome in the samples [61]. Culture-dependent methods, which involve microbial isolation on nutrient-rich microbiological media under specific revolutionized growth conditions, are important to determine microbial physiology and genes and screening for plant growth-promoting traits [62].

Conversely, this technique is laborious, revealing detailed microbial diversity and networking in econiches. Also, the proliferation of undesirable microorganisms on the cultured plates, which compete for nutrients needed by the desirable microorganisms, has been identified as a major challenge when isolating endophytic microbes by culturing methods [63]. Hence, the application of culture-independent methods is profound in characterizing yet-to-be cultured microorganisms. Authors Alain and Querellou [64], Torsvik and Ovresas [65], and Afzal, Shinwari, Sikandar, and Shahzad [63] stated that culturable bacteria represent about 0.0001–1% of the total endophytes in plants. Hence, the interest of researchers on purposeful research design should be considered before selecting a method for isolating endophytic microbes.

Endophytes can be cultured on agar plates, and then microbial DNA can be extracted before carrying out polymerase chain reaction (PCR). Garcias-Bonet, Arrieta, de Santana, Duarte, and Marbà [66] employed a commercial DNA extraction kit specific for plant DNA extraction to extract microbial endophytic DNA and used primer meant for the bacteria domain to carry out the PCR procedure. However, it should be noted that when amplifying a specific region of bacteria DNA, the mitochondria and chloroplast DNA found in plants may have a close resemblance to that of endophytes; hence, this method might not be too appropriate. In this light, next-generation sequencing is recommended without denaturing gradient gel electrophoresis (DGGE) analysis. Piccolo, Ferraro, Alfonzo, Settanni, Ercolini, Burruano, and Moschetti [67] demonstrated the use of fluorescence in situ hybridization (FISH) technique in studying endophytic microbes. However, this can only be done in the natural habitat, thus making the laboratory isolation complicated.

On the other hand, Ikeda, Kaneko, Okubo, Rallos, Eda, Mitsui, Sato, Nakamura, Tabata, and Minamisawa [68] developed a procedure to enrich bacterial cells when isolating unculturable endophytes from the stem of a soybean by fractionalizing the homogenated soybean stem. This method was achieved by differential centrifugation and Nycodenz density gradient centrifugation. This method proved effective compared to when DNA was isolated from the soybean stem due to the higher intensity and number of amplicons of the bacteria when the efficiency of the bacteria cell was fortified using ribosomal intergenic spacer analysis. Equally, Lundberg, Yourstone, Mieczkowski, Jones, and Dangl [69] also worked on an improved technique for 16S ribosomal rRNA sequencing, where unique template molecules were tagged before PCR by mapping amplicon sequences (to their original templates), which help to prevent error and bias arising from the amplification process. This method uses a base pair sequence with a higher temperature (melting) than the primer set, which is designed to attach to the host’s DNA.

The culture-independent methods are more advanced due to attention drawn to them which facilitated more research to improve them. For instance, a modern analytical approach has been documented to advance the science of the plant microbiome [70]. The use of combined stable isotope probing (SIP) and nanoscale secondary ion mass spectrometry techniques (NanoSIMS) coupled with advanced Raman spectroscopy-based single cell–based methods have been envisaged in studying plant microbiome in situ and to determine their biological functions in the bioremediation of complex pollutants from metal-polluted soil [71]. More importantly, the specific metabolic functions of endophytic microbes can be better understood by combining SIP with other molecular methods, such as qPCR, finger printing, and cloning.

Dos Santos and Olivares [72] reported the use of microcosm combined with bacteria stocks as a reference to determine bacteria assemblage in the root of plants and their plant growth-promoting potential. Also, Hartman, van der Heijden, Roussely-Provent, Walser, and Schlaeppi [73] reported a microcosm approach in elucidating the bacteria diversity and function in the root of red clover. Furthermore, Hartman, van der Heijden, Roussely-Provent, Walser, and Schlaeppi [73] revealed a significant reduction in the growth of red clover upon mono-inoculation with *Flavobacterium* compared to the co-inoculation of red clover with root microbiome, which enhanced plant growth by reducing the negative effect of mono-inoculation of red clover with *Flavobacterium*. Finally, a microcosm study performed by Eldridge, Travers, Val, Ding, Wang, Singh, and Delgado‐Baquerizo [74] reported diverse microbiome and their functions on 15 plant species growing in terrestrial habitats to reveal the preference of plant-associated microbes and their importance in plant germination. It will be interesting to fashion out how these modern approaches can be employed in the science of endophytes to better understand endosphere biology.

Different molecular approaches exist for the identification of endophytic bacteria and the combination of recent molecular approaches, such as genome sequencing and metagenome. The use of DNA extracted from the root of plants can be employed in unraveling microbial community structure and functions in soybean. The extracted DNA from plant tissues after surface sterilization with water, hypochlorite, or ethanol for endophytic studies might contain a certain proportion of plant DNA, which is needed to be depleted using appropriate sequencing techniques and plantforms (e.g., Illumina, PacBio, and DNA fingerprinting). Many techniques exist for DNA fingerprinting. These include restriction fragment length polymorphism (RFLP), simple sequence repeat (SSR), terminal-RFLP, rapid amplified polymorphic DNA, amplified fragment length polymorphism, inter-SSR, single-stranded conformation polymorphism, and DGGE [75]. The analysis of diverse plant microbiomes based on genetic composition can be achieved by the real-time polymerase chain reaction, FISH, automated version of ribosomal intergenic spacer analysis, terminal restriction fragment length polymorphisms, and DGGE, and phospholipid and fatty acid have also been documented [72, 76–78].

It is noteworthy to understand the use of molecular methods in identifying yet-to-be microbial endophytes by using appropriate methods to maximally recover endophytic DNA after the extraction process. The advent of PCR-based approaches in the Plant-Microbial Genome Project has provided vast advantages and opportunities for the detection, multiplication, quantification, and synthesis of copies of DNA in large amounts, differentiated from one another [79]. PCR techniques have been widely employed for the detection of diverse genes responsible for microbial functions [80]. The PCR and DNA sequencing aims at measuring the presence, taxonomy, and functions of plant microbiome from various samples, although, despite the importance of these techniques, there are limitations surrounding the PCR amplification process and DNA sequencing, mostly when extracting DNA from plant samples. The limitations include (i) contamination during the DNA extraction for PCR reaction and library preparation which may affect the DNA integrity, resulting in result errors and false outcome; (ii) primers’ design which require some previous sequence information; and (iii) the specific PCR product obtained during amplification process may be altered from one microbe to another based on non-specific binding of primers to other identical targeted sequences [81]. Piccolo, Ferraro, Alfonzo, Settanni, Ercolini, Burruano, and Moschetti [67] demonstrated the use of FISH technique in studying endophytic microbes; however, this can only be done in the natural habitat, thus making the laboratory isolation complicated. Furthermore, addressing these limitations specific to sequences may help devise approaches for the normalization of sequenced data to reveal microbial composition in its entirety. The use of PCR coupled with other sequence-based approaches is promising with more insights into plant microbiome gene combinations [71].

The advent of advanced molecular techniques for endophytes’ identification has succeeded in DNA fingerprinting, for instance, the use of omics approaches where DNA is retrieved from bacteria to evaluate the diversity, functions, genes, metabolites, transcripts, and proteins with the aid of next-generation sequencing. The DNA fingerprinting methods have been overtaken by more technical procedures, such as metagenomics which involves DNA extraction from the total bacteria population using next-generation sequencing [82]. This method has proven to better unravel the total endophytes from plant tissues compared to the fingerprinting techniques. Aside from omics approaches, the use of microscopy techniques, epifluorescence light microscopy, bright-field light microscopy, interferential and differential contrast light microscopy, scanning electron, and transmission electron microscopy in determining visual evidence of microbial colonization patterns in plants, has been documented [72, 76, 83].

The use of culture-independent techniques, which involved DNA/RNA extraction from environmental samples coupled with omics approaches, has revolutionized the science of endophyte microbiology in generating large sequence datasets. This next-generation sequencing approach involving no DNA cloning has been employed to unveil the community structure, diversity, taxonomic and functional profiling, metabolites, and metabolic pathways of the plant microbiome [72]. So far, the few research efforts utilizing next-generation sequencing from soybeans and other food crops revealed their taxonomic and functional attributes of endophytes in different plant species (Table 2).

**Table 2:** Shotgun metagenome findings of endophyte from legumes and food crops

Host plant	Main findings	Recommendation(s)	References
**Sunflower**	Assessment of endophytic bacteria in the root of growing sunflower in South Africa	Future isolation of identifiable bacteria in determining their functional traits for plant nutrition	[61]
**Maize**	Shotgun metagenome approach in determining the diversity and functional genes maize-root associated endophytic bacteria	The need to understand the mechanisms and colonization patterns of bacteria in the plant endosphere. Exploration of bacterial with functional genes in agricultural productivity	[158]
	Assessing maize seed endophytic bacteria community structure of genetically related genotypes	Suggestions for future study on endophytic bacteria in maize of different genotypes and mechanisms of transmission	[195]
**Soybean**	Endophytic bacteria in soybean endosphere	Exploration in agricultural biotechnology	[159]
	Dynamics of endophytic bacteria diversity in the root nodule of soybean	Further studies on the dynamics and functions of bacteria in the root nodules for improved soybean varieties	[107]
**Sorghum**	Genome and metagenome profiling endophytic bacteria in sorghum	Future exploration of sorghum associated bacteria in a similar experiment for optimal use in agricultural productivity	[196]
**Tomatoes**	Understanding the population and functions of endophytic bacteria in the pathogen-infected and non-infected tomato roots	Strategic measures for future exploration of endophytic bacteria in the control of plant-parasitic nematodes for improved yield	[197]
**Rice**	Insights into functional attributes of rice endophytic microbiome using shotgun metagenome approach	Designing genomic systems in studying the endosphere community in the root of rice to detect copious bacteria as a model trait, which can be explored in the bioremediation process, disease control, and plant nutrition	[198]
	Illumina sequencing of diverse rice associated endophytic bacteria	Further studies on the unexplored endophytic bacteria in rice genotypes and the influence of environmental factors on plant microbiome diversity	[199]
**Wild soybean, yellow pea bush, sorghum**	Determining the diversity and function genes of endophytic bacteria in the salt-tolerant legume and non-legume plants	The need to explore legume and non-legume crops associated with bacteria for salt stress alleviation in plants	[29]
**Tree peony**	Endophytic bacterial diversity in the root and leaves of tree peony using next-generation sequencing	Further studies on the root exudation influence on bacteria diversity as a model in endosphere biology	[200]
**Mung bean**	Differences in the root nodule endophytic bacteria diversity as revealed by Illumina sequencing in growing mung beans at different locations	Exploration of endophytic bacteria as bioinoculants for mung bean production	[201]
**Chickpea**	Assessing diverse endophytic bacteria with notable functions in Chickpea	Harnessing endophytic bacteria as bioinoculants for plant nutrition	[202]
**Wheat**	Investigating the influences of diazotroph on the composition and function of microbial communities associated with the rhizosphere and endosphere of wheat	Exploration of nitrogen-fixing diazotroph *Paenibacillus triticisoli* BJ-18 as bioinoculants, which underline the diversity and functional traits of plant microbiome for improving plant growth under natural field conditions	[203]

Furthermore, addressing these limitations specific to sequences may help devise approaches for the normalization of sequenced data to reveal microbial composition in its entirety. The use of PCR coupled with other sequence-based approaches is promising with more insights into plant microbiome gene combinations [71]. The combination of recent molecular approaches, such as genome sequencing and metagenome using DNA extracted from the root of plants, can be employed in unraveling microbial community structure and functions in soybean. More importantly, the specific metabolic functions of endophytic microbes can be better understood by combining SIP with other molecular methods, such as qPCR, finger printing, and cloning.

The advancement in plant microbiome studies has revealed certain traits, which mediate their functions, such as secondary metabolites, genetic information, proteins, and transcripts using culture-dependent and culture-independent techniques [84–86]. Modern approaches to studying diverse endophytic microbes and functions are being employed to understand the colonization pattern for plant–microbe interactions based on host specificity and signaling networking for microbial communications linked to root exudation [87]. The genes involved in flagellation, chemotaxis, motility, and biofilm formation has been reported in many bacteria strains, which facilitate their attachment/adherence, penetration, and colonization in the host plants [88, 89]. The host plants’ specific signal-networking and plant–microbe communications can reveal how microbes exhibit mutual relationships and antagonistic toward the phytopathogens by triggering host immune responses [12]. Aside from genes involved in beneficial bacterial colonization, other genes have also been documented to partake in microbial biological processes. For instance, the genes involved in carbohydrate metabolism, phytohormone synthesis, secretion systems, biocontrol activity, and oxidative stress identified in the genome of endophytic bacteria from sunflower, apricot, and poplar that are important in agriculture, biotechnology, and industry have been documented [90–92].

In line with the aforementioned approaches and conventional techniques, studying the plant microbiome can be easier. Hence, it is recommended to compare the different recovering or identifying endophytes. This would assist in selecting the best method to use to identify endophytic microbe from plant samples. On the other hand, both the culture-dependent and culture-independent methods of endophyte analysis can help have a broader view of the diversity and population of plant endophytes and their functional attributes in the ecosystem. Briefly, the advantages and disadvantages of the techniques and approaches employed in the study of plant-associated microbes are highlighted in Table 3.

**Table 3:** Some techniques and approaches of studying plant-associated microbes

Culture-dependent			Culture-independent		
	Advantages	Disadvantages		Advantages	Disadvantages
1. Culturing by plating techniques	a. Easy to perform microbial isolation on nutrient-rich microbiological media under specific growth conditions b. Effectiveness in obtaining pure cultures c. Easy to characterize microbial morphology, phylogeny, physiology, and biochemistry d. Easy to screen microbial metabolites e. Easy to evaluate microbial populations f.f.Easy to extract microbial genetic materials (DNA) g. Low cost	a. It is laborious b. Difficulties in assessing diverse microbial communities due to the varied growth parameters required for culturing c. The need for skillful experts and professionals in the sequence analysis d. Proliferation of undesirable microorganisms	1. Molecular based techniques	a. Easy to extract microbial genetic materials (DNA/RNA) b. Revealing of the total microbiome in the samples c. Identifying the role of microbes in various biological processes d. Provide detailed information on the microbial taxonomy profiling, functions, metabolites, metabolic pathways	a. Presence of plant DNA as contaminants alongside with the endophytic DNA b. High cost of purchase of DNA extraction kits, primers, genomic sequencing c. Extra cost of depleting host DNA for sequencing d. The low efficiency of endophytic DNA after extraction and amplification of the 16S rRNA e. The sometime result of small amounts of endophytic DNA after DNA extraction f.f.Require skillful experts and professionals in the sequence analysis
			2. PCR techniques and DNA sequencing (Detection of microbial genes)	a. Easy to measure the presence, taxonomy, and functions of plant microbiome	a. Contamination during the DNA extraction for PCR reaction b. Libraries preparation may affect the DNA integrity, thus resulting in result errors and false outcome c. Primers design which require some previous sequence information d. The specific PCR product obtained during amplification process may be altered from one microbe to another based on non-specific binding of primers to other identical targeted sequences
			3. Microscope techniques	a. Easy to visualize detailed microbial structures and colonization patterns	b. It can only be used in the presence of light c. It is cumbersome d. High cost of purchase e. Large in size f. Highly sensitive to vibration and external magnetic fields
			4. Stable isotope probing (SIP)	a. Identification of specific metabolisms within a microbiome b. Interrogation of microbial communities in environmental samples	–
			5. Microcosm	Easy to access the effect of toxic compounds under controlled conditions on natural microbial communities	–

## Complexity of Plant Microbiome in Plant Ecosystem

**Fig. 1:**
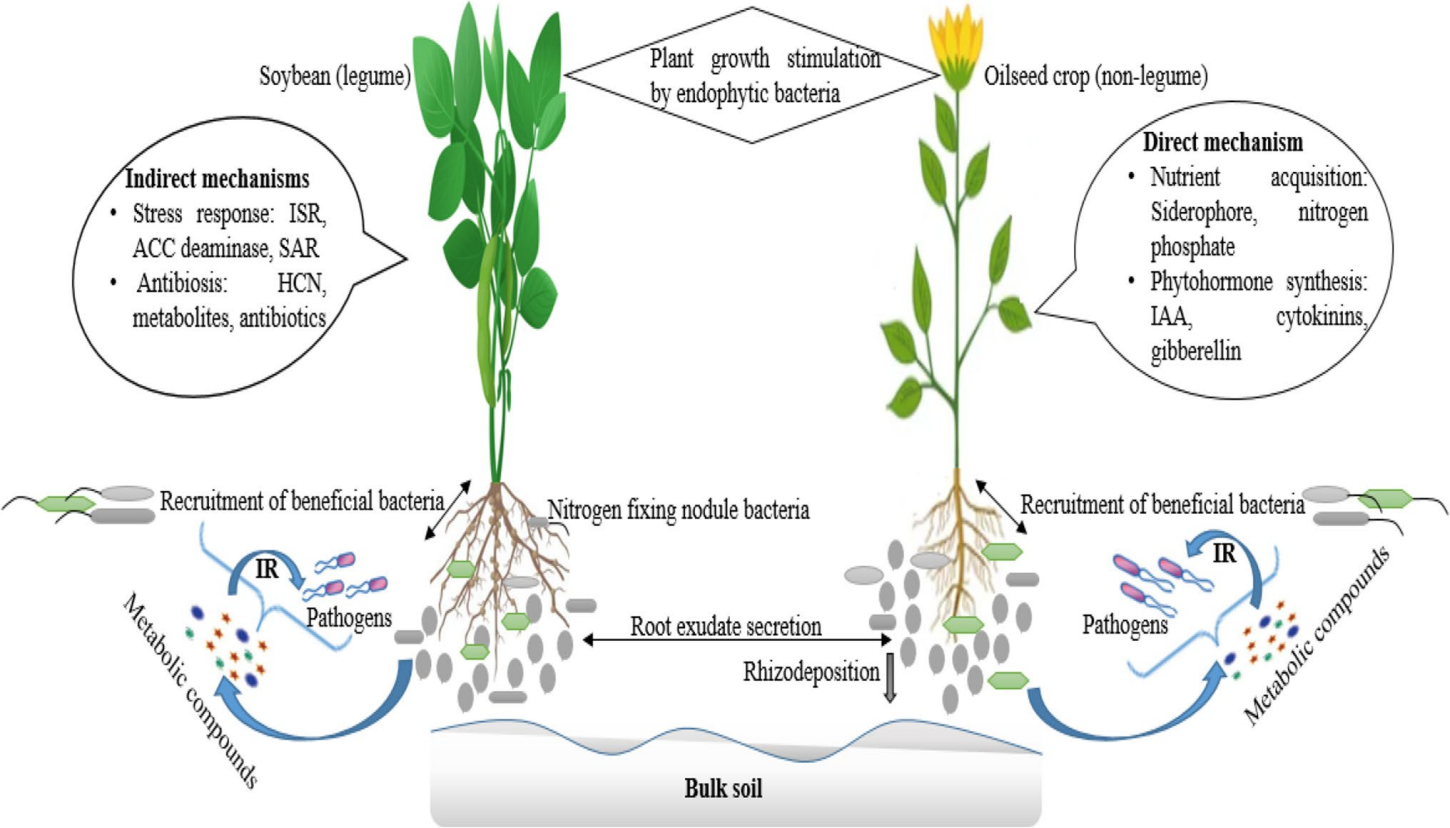
Endophytic bacteria recruitment mechanisms and benefits in plant growth promotion. Key: IR, induced resistance; HCN, hydrogen cyanide; SAR, systemic acquired resistance

The microbes recruited into the plant endosphere and those inhabiting the external root environment contribute to plant growth in diverse ways, as shown in Fig. 1. Reports by Ku et al. [93] showed root surface and hair colonization by an endophytic bacterium, *B. cereus*, in Chinese cabbage, soybean, and wheat, with evidence in understanding the mode of actions of plant microbes and how they influence plant growth. Aside from endosphere and rhizosphere research findings, fewer studies have documented the microbiome inhabiting the antosphere, caulosphere, carposhere, and spermosphere. Research into microbiome in the plant environments, such as rhizosphere, root, seed, and stem, have been documented, and their possible use in agricultural biotechnology is profound.

For instance, Kumawat et al. [94] reported an increase in the growth, symbiotic efficacy, nutrient acquisition, and yield of soybean co-inoculated with endophytic *Pseudomonas oryzihabitans* and *Bradyrhizobium* spp. Also, an increase in the crop yield, oil content, antioxidant content, seed quality, carbohydrates, and chemical composition (protein and lipid) of soybean inoculated with endophytic *Bacillus amyloliquefaciens* has been reported by Sheteiwy et al. [23], which suggests their future exploration as bioinoculants in growing soybean under drought stress. Because plants harbor diverse number of microorganisms, the better understanding of their complexity and functional traits will help unraveled their biological activities.

### Rhizosphere and Bulk Soil Microbiome

The rhizosphere (plant microhabitats) represents soil regions closer to the plant root environment [95]. The rhizosphere is often referred to as a “hotspot” for microbial activities due to the excess release of root exudates, which supply the required energy for microbial metabolic activities [96]. The response of soil microbes to the diverse chemical compounds and varied soil parameters, which favors soil microflora, can be an indicator for selecting them over others. The shaping of the rhizosphere microbiome can be a function of the quantity of exudate released from one plant to another. Some examples of secondary metabolite organic compounds include amino acids, phenols, organic acids, sugars, siderophores, polysaccharides, etc. When released from plant roots, it influences a higher microbial population in the rhizosphere than in bulk soil [97].

Bulk soil is the soil that is equidistance away from the rhizosphere region without root penetration [98]. The microbial inhabitant in the bulk soil can be less in diversity due to the fewer organic compounds than the rhizosphere soil inhabitants with identical species. Some examples of bacteria occupying the rhizosphere root environment include *Bradyrhizobium diazoefficiens, Bacillus subtilis, B. velezensis*, etc. [99–101]. High microbial colonization, diversity, and activities are easily mediated by rhizodeposition, reducing compared to the adjacent or bulk soil [102].

Usually, the variations in the rhizosphere microbial communities in soybean and other food crops can be linked to the geographical locations, growing seasons, crop rotation, plant growth stages, cultivars, farm practices, etc. The identification of diverse bacterial phyla, such as Acidobacteria, Actinobacteria, Bacteroidetes, Chloroflexi, Gemmatimonadetes, and Proteobacteria from rhizosphere soils under the different growing conditions and soil types, has been reported with greater influence on the bacterial diversity [103, 104]. Hence, there is a need for further research to ascertain if the microbes present in soybean across multiple locations are different, since there is less information of the soybean rhizosphere microbial communities.

Due to the nodule formation in the root of soybean, the endosymbiotic relationship with nitrogen-fixing bacteria can be an advantage in releasing excess quality, and quantity of root exudate different from non-nodulating plants to help establish discreet microbial biomass in the rhizosphere [18]. High-throughput sequencing in determining the rhizosphere bacterial and fungal communities of rapeseed have revealed varied operational taxonomic units at seedling, flowering, and maturity stages [105]. The assessment of a bacterial community in rapeseed using a molecular ecology network with random matrix theory showed bacteria genera, such as *Rhizobium*, *Flavobacterium*, and *Pseudomonas*, at the network level [106].

Interestingly, research on rhizosphere microbiome with the view of mapping out strategies for their incorporation into agriculture has been emphasized in recent times [107–109]. Nevertheless, the presence of pathogens may influence rhizosphere microbes in many ecological processes. Furthermore, the source of rhizosphere microbes is important as most of them may be introduced into the soil through seed planting [110].

### Seed Microbiome

Aside from the rhizosphere microbiome [111], research advancements have shown microbial composition on the surface and internal tissue of seeds [76, 112] can be beneficial or pathogenic. The beneficial microbes influence seed growth at pre-germination, germination, flowering, and maturation stages [113]. The recruitment of microbiome in seeds can be achieved by vertical (from the mother plant) or horizontal (environment) transmission [114]. Vertical transmission of seed endophytes is believed to originate from the leaves and flowering parts. Upon planting into the soil, the seed undergoes imbibition, which enables them to absorb soil nutrients and then germinate. During the imbibition process, the release of metabolic compounds in the spermosphere region, i.e., soil-seed environment, creates an attractive environment for the soil microbes to compete with the natural soil pathogens [115]. At this stage, the seed microbiome infiltrate or release to the soil environment via horizontal transfer.

Seed endophytic bacteria and their mode of transmission enabled them to occupy diverse niches, such as the pericarp, seed embryo or cotyledon, and endosperm [116]. The transmission of seed endophytes may differ depending on the organ location. For instance, endophytic in the pericarp are horizontally transmitted, while those colonizing the endosperm and embryo are easily transmitted by vertical processes [117]. More research should be done on soybean to understand how endophytes are transferred in their root region.

Microscopy and high throughput sequencing approaches have been employed to characterize seed microbiome, especially endophytic bacteria in some leguminous plants [118]. Sánchez-López et al. [119] reported dominant endophytic bacteria phyla, Proteobacteria, Firmicutes, Chlamydiales, and Bacteroidetes while investigating endophytic bacteria in the seed of Low Rattlebox. Information on the microbial community structure of endophytic bacteria in the seed of soybean and other leguminous food crops using high-throughput sequencing are scanty in literature. Consequently, differentiating seed endophyte and soil microbiome are still less understood. Also, seed endophytes can be found in other plant parts via infiltration from the rhizosphere to the above ground level. Interestingly, the synergistic cooperation between the soil microbes and seed endophytes has contributed to plant health and nutrition [120].

### Root and Shoot Microbiome

The root and shoot form a key component in the study of the plant microbiome [71]. The microbes found in the root and shoot can be less in number compared to the higher microbial profiling in the rhizosphere due to the nutrients and exudate secretion attributes [96, 121]. The detection of genes involved in bacteria attachment due to specialized cell organelles, such as fimbriae, flagella, and pili in the plant surface, assisted bacteria adhesion to the cell surfaces to form a biofilm [122]. The plant-bacteria interaction and transient within the plant tissue can result due to a rise in water flux during transpiratory processes in plants. Across the plant parts, the presence of the targeted microorganisms may be influenced by the organ location and accessibility to plant nutrients [123].

For microbes to efficiently colonize the host plant, the line of mode of actions involved includes (i) adherence to the root surface, (ii) multiplication, (iii) invasion from the external root environment, and (iv) colonization [124]. After the colonization process, the movement of endophytic microbe from the belowground to the shoot through microbial networking can be achieved. The type and quantity of nutrients available in the plant endosphere can modulate the extent of bacteria diversity. Adeleke et al. [61] and Jie et al. [125] reported diverse endophytic bacteria phyla Chloroflexi, Nitrospirae, Planctomycetes, Palescibacteria, Acidobacteria, Actinobacteria, Cyanobacteria, Saccharibacteria, Firmicutes, Gemmatimonadetes, Bacteroidetes, and Proteobacteria from the root of sunflower and soybean. Recently, endophytic bacteria genera *Bacillus*, *Staphylococcus*, *Serratia*, *Stenotrophomonas*, *Pseudomonas*, *Enterobacteria*, and *Erwinia* from healthy rapeseed has been reported as part of the shoot microbiome [25]. Endophytic bacteria in the external root environment are usually higher compared to the internal part of roots. In the findings by Adeleke et al. [61], the authors reported a dominant and high bacteria population in the root of growing sunflower compared to the stem due to the agricultural practices, geographical locations, plant type, organ location, etc., which contribute to the bacterial diversity.

The reason for microbial differences in the rhizosphere, endosphere, and phyllosphere can be biological, chemical, or physical factors, which may exert selective pressure on endophytic bacteria to infiltrate the root endosphere [126]. The endophytic microbiome tends to adjust to a plant environment with stable biomass, while the rhizosphere microbiome may vary due to niche complexity. Acknowledging the fact that plants harbor a multifunctional microbiome in the root and shoot can be a pointer to understanding factors that modulate the shape of the microbiome in plants.

## Plant Growth Stimulation Attributes of Endophytic Bacteria

Beneficial plant microbiome helps in sustaining the ecosystem [100]. The ecological services range from plant growth promotion, pathogen control, phytoremediation, biofertilization, and abiotic stress mitigation to human safety [86, 127]. In recent times, the multifunctional attributes of endophytic microbes as plant growth stimulators and bioinoculants promise to revolutionize agriculture without negative ecological effects [128]. Also, the role of endophytic microbes in agricultural biotechnology has been focused on; yet, research is still ongoing to meet zero ecological threats for maximum food production [129]. Exploration of endophytic resources to provide alternative measures in ensuring a safe environment and sustainable agricultural productivity have been emphasized due to the negative impact of chemical fertilizers on the ecosystem [130].

From the multifaceted application perspective, the mechanisms employed by endophytic microbes immensely contribute to plant growth and health [22]. Microbes employ direct or indirect mechanisms in sustaining plant growth and health [131]. The core attributes of endophytic microbes in enhancing plant growth include nutrient acquisition and mineralization, phosphate solubilization, nitrogen fixation, siderophore and enzyme production, and synthesis of growth hormones, such as indole-3-acetic acid, gibberellic acid, and abscisic acid, while indirectly, ACC deaminase, exopolysaccharide, and hydrogen cyanide production by endophytic microbes contribute to plants survival under drought stress [132]. All the aforementioned processes, specifically, have been screened from endophytic microbes associated with sunflower and soybean [15, 40]. In addition, the suppression of phytopathogens through the induction of systemic resistance and antibiosis activities of endophytic microbes boost plant immunity against soil and host invading pathogens [133]. Also, findings by Zhao, Xu, and Lai [9] reported high inhibitory activity of soybean nodule endophytic bacterium *Acinetobacter calcoaceticus* against pathogenic fungus *Phytophthora sojae* due to their close association with the root of the plants.

Endophytic microbes are said to deliver effectively in enhancing plant growth due to their close interaction, colonization, less composition in plants, and non-exposure to harsh environmental conditions [134]. These attributes make endophytic studies interesting compared to the rhizosphere microbes. The synergistic effect of nodule endophytic bacteria, *Pseudomonas aeruginosa* (LSE-2) and *Bradyrhizobium* sp. (LSBR-3) from soybean, has been investigated as a source of bioinoculants and biofertilizers due to their root colonization potential through molecular crosstalk, which supports plant growth and nutrition [135].

Some endophytic microbes solubilize phosphate in natural form by producing organic acids, which lower soil pH and chelate iron for easy phosphate assimilation by plants in soluble form [136]. The ability of endophytic bacteria to produce phosphatases also helps in the mineralization of organic phosphorus [137]. In vitro screening of phosphate-solubilizing endophytic bacteria has been investigated from soybean, sunflower, and rapeseed [138–140]. For example, *Acinetobacter calcoaceticus*, *Ochrobactrum haematophilum*, *B. panacihum*, *Bacillus subtilis*, *B. australimaris*, *B. thuringiensis*, *B. zhangzhouensis*, and *Lysinibacillus pakistanensis* have been isolated from leguminous crops [9, 18, 30]. Kenasa, Nandeshwar, and Assefa [141] reported the identification of cowpea root endophytic bacteria, *Pseudomonas putida*, and *Bacillus subtilis* phosphate producers in their study. Also, a study by Yasmeen and Bano [142] reported an increase in soybean yield co-inoculated with phosphate-solubilizing bacteria, *Rhizobium* and *Enterobacter*.

The rhizobacteria in the root nodule of leguminous plants naturally fix nitrogen in the soil, which is needed for plant nutrition [18]. The nitrogen fixation potential of endophytic bacteria in the root nodules of leguminous crops, effectively, has enhanced the nitrogen pool in soil deficient in nitrogen supply [143]. The nitrogen fixation by endophytic bacteria may differ compared to rhizobacteria found in the root of legumes [144, 145]. Interestingly, exploration of the endophytic bacterium *Gluconacetobacter diazotrophicus* with exceptional nitrogen fixation in plants has long been reported in reclaiming nitrogen loss in the soil [146].

The ability of endophytic bacteria to produce siderophores also plays a major role in plant health sustainability [147]. For instance, biocontrol activity which limits iron supply to the pathogens, heavy metal reduction, and induction of systemic resistance can be linked to the siderophore compounds, i.e., catecholate and hydroxamate, produced by endophytic bacteria [148]. Diverse endophytic bacteria associated with soybean have been reported as siderophore producers [15]. Bhutani et al. [18] and Maheshwari et al. [149] reported siderophore-producing endophytic bacteria strains from legumes. The suppressive and biocontrol activity of endophytic *Burkholderia contaminans* against *Macrophomina phaseolina* causing root rot, stem rot, seedling blight, damping off, and charcoal rot in jute due to siderophore biosynthesis has been reported [150]. Since the presence of nitrogen-fixing and siderophore-producing bacteria has been established in soybean, other functions of these bacteria should be further studied.

Similarly, phytohormones, such as ethylene, IAA, cytokinins, and gibberellin, modulating plant growth via diverse pathways are evident in endophytic microbes [9]. Notably, IAA biosynthesis facilitates root development, which enables plants to absorb nutrients and water from the soil [151]. Tryptophan, which serves as a precursor for IAA production by endophytic microbes in a growth media, helps differentiate IAA-producing bacteria from non-IAA-producing bacteria [152]. Evidence of IAA and other phytohormones, such as gibberellin, and cytokinin production by endophytic bacteria to enhance plant growth, have been documented [63, 153]. Some endophytic bacteria, which produce 1-aminocyclopropane-1-carboxylate (ACC), a precursor for ethylene production, contribute to plant growth and are resilient to drought stress [154]. The ability of endophytic bacteria to circumvent the effect of pathogens by producing jasmonic acid, antibiotics, salicylic acid, volatile compounds, siderophore, and lipopolysaccharide, elicit induced systemic resistance, and abiotic stress amelioration in the host plants [155].

The actual mode of actions employed by endophytic bacteria in oilseed crop soybean is yet to be fully understood. Similarly, the biosynthesis and metabolism of reacting molecules as precursors for the synthesis of novel metabolites or enhancing already identified metabolites are poorly understood. The synthesis of secondary metabolites, such as alkaloids, terpenoids, phenols, organic acids, and flavonoids, which induce antibiosis, can be achieved by endophytic microbes specific to the host plants [156]. Some examples of purified secondary metabolites produced by endophytic bacteria from some economic plants with related biological functions are presented in Table 4. Information relating to secondary metabolites sourced from endophytic bacteria associated with soybean is less documented in the literature. Hence, research focusing on secondary metabolites from endophytic bacteria associated with soybean and their exploration will further reveal their bioprospecting in plant disease management.

**Table 4:** Secondary metabolites originated from endophytic bacteria in plants and their bio-properties

Plants	Bioactive compound	Endophytic bacteria	Purification method	Biocontrol activity	References
Cannabis	Cotinine, 4-acetamidobutanal, S-2charCodeAt443, l-2-aminoadipate, 4-acetamidobutanoate, l-ornithine, l-saccharopine, 3-O-methyldopa	*Serratia marcescens*, *Enterobacter cloacae*, *Paenibacillus* spp.	LC–MS/MS	Antimicrobial	[204]
Chickpea	Fengycin, surfactin, iturin	*Bacillus siamensis*	LC.MS	Antifungal	[205]
Fingerroot	2′,7-Dihydroxy-4′,5′-dimethoxyisoflavone, 7-methoxy-3, 3′,4′,6-tetrahydroxyflavone, fisetin, hydroxydaidzein, naringenin, 3′-xenognosin B	*Staphylococcus aureus*, *Streptomyces* spp*.*, *Bacillus cereus*, *Bacillus subtilis*	CC, TLC	Antibacterial	[206]
Aquatic plant	1,4-Diaza-2,5-dioxo-3-isobutyl bicyclononane	*Streptomyces coeruleorubidus*	GC–MS	Antifungal, antioxidant, and cytotoxicity activities	[207]
Dabieshan white pine	Fungichromin	*Streptomyces* spp.	HPLC	Antifungal	[208]
Chinese skullcap	FengycinAB, surfactin,	*Bacillus amyloliquefaciens*	HPLC–MS	Antimicrobial	[209]
Chilli	2,5-Piperazinedione, 3,6-bis(2-methylpropyl), pyrrolo[1,2-a]pyrazine-1,4-dione hexahydro-3-(2-methylpropyl), 1,2-benzenedicarboxylic acid, diisooctyl ester, pyrrolo[1,2-a]pyrazine-1,4-dione, hexahydro-3-(phenylmethyl), pyrrolo[1,2-a]pyrazine-1,4-dione, hexahydro	*Bacillus subtilis*	GC-HRMS	Antibacterial	[210]
Bellflower	Syringin, lobetyolin, atractylolide III	*Pseudomonas nitroreducens*	HPLC–UV	Antimicrobial	[211]
Cuneate Wedge Shell	Astaxanthin	*Pontibacter korlensis*	HPTLC, HPLC	Antibacterial, antioxidants	[212]
Summer snowflake	Tazettine, lycorine, pseudolycorine, acetyl pseudolycorine	*Bacillus* spp.	NMR, LC–MS	–	[213]
Golden thread herb	Berberine	*Burkholderia* spp., *Microbacterium* spp.	–	Anti-tumor, anti-inflammatory	[214]
Garden thyme	bis (2-Ethylhexyl) phthalate, 1,3-dimethyl-, p-xylene dibutyl phthalate, etracosane	*Bacillus subtilis*	–	Antimicrobial activity	[215]

*HPLC* high-performance liquid chromatography, *LC–MS* liquid chromatography-mass spectrometry, *NMR* nuclear magnetic resonance, *CC* column chromatography, *TLC* thin-Layer chromatography

The biomolecules sourced from endophytic bacteria stand promising in agriculture, environment, industry, and human safety. Hence, genomic insights into plant microbiome aim to reveal their functions and activity in plant physiology and metabolism. Additionally, it is imperative to unravel soybean-associated endophytic bacteria’s biological functions and physiological attributes, using culture-dependent and culture-independent techniques to identify secondary metabolites in the bacteria genome [24]; making information available on secondary metabolites produced by endophytic bacteria will help find a solution to diverse agricultural problems.

## Current Status of Plant Microbiome Collaborative Research

The interdisciplinary synergies among researchers in studying plant–microbe interactions continue to progress. Research efforts to study and explore endophytic bacteria from the leguminous crop as bioinoculants for plant growth and sustainable ecosystem have increased tremendously with driven biotechnological advances and low-cost analysis [124]. Interestingly, the commercialization of endophytic bioinoculants is possible in sustainable agriculture [128]. The computational knowledge about next-generation sequencing and other innovative techniques have informed scientists with accurate information on microbial diversity and related genes [109].

Better still, there is a need to develop robust bioinformatics tools and analytical techniques with the existing technologies to generate microbiome data as a guide for further experiments. Adeleke et al. [40] and Adeleke et al. [157] reported the genomic characterization of plant growth-promoting endophytic bacteria, *Bacillus cereus* T4S and *Stenotrophomonas maltophilia* JVB5 as copious sunflower growth enhancers. Furthermore, effort on the use of these endophytic bacteria as biocontrol agent against phytopathogens is expected to be investigated in the future studies. Employing this approach by ecologists, environmental and computational scientists, microbiologists, agriculturists, and industrialists aim to provide insights into plant microbiome research as a reference for further studies. Furthermore, understanding the dynamics and role of endophytic microbes in plants using up-to-date techniques and bioinformatics tools, however, can help develop multiple strategies in understanding their functions in diverse fields, such as agriculture, ecology, medicine, forensics, and exobiology.

The dominant bacteria phyla, Actinobacteria, Firmicutes, Proteobacteria, Bacteroidetes, and Chloroflexi in the root endosphere of food crops, such as maize, cowpea, sorghum, sunflower, soybean, have been reported using culture-independent techniques [61, 158, 159]. Yet, there is a need to investigate further using appropriate techniques to access plant growth-promoting endophytic bacteria in different legumes and other food crops under different climatic conditions. Hence, identifying these bacteria for bioinoculants formulation can serve as a pointer to achieving ecofriendly agriculture sustainably.

## Conclusions

This review evaluates endophytic bacteria in soybean and other food crops. The bioprospecting of these bacteria enhances their potential for sustainable yield enhancement. Soybean was discussed as a reference crop for oilseed crops due to its economic importance, high yield, and nutritional value. Soybean harbors some endophytic microbes important in agriculture. Beneficial endophytic microbes inhabiting different parts of the plants can potentially contribute to the growth of soybeans and other food crops. For instance, root nodule bacteria and endophytic bacteria enhanced nitrogen fixation in soybean, which promotes their yield and other yield parameters, enhance immunity, and boost plant defense against diseases. However, the root endophytes are emphasized because of high metabolic activities occurring below ground level due to the high quantity of metabolite secretion, which contributes to plant physiological functions.

Different conventional and molecular techniques have been employed in the past to unravel endophytic microbes in some plants; nevertheless, each method comes with shortcomings. For instance, some endophytes can be difficult to culture on media despite their viability, such that culturing method can only unravel a lesser percentage (i.e., 0.1%) of endophytic populations. Hence, the advancement of endophytic microbes discovery using molecular techniques has proven more promising, although, with diverse challenges. Extracted endophytic bacteria DNA might contain traces of plant DNA, the chloroplast, and mitochondria DNA, which are identical to the targeted endophytic bacteria DNA [160]. Host depletion techniques have been employed to remove a substantial amount of plant DNA that might be present in the DNA extracted from the plant tissues. Conversely, the use of fluorescence in situ hybridization (FISH) is inefficient, because it can only be carried out in a natural habitat.

Oilseed crop soybean is economically important due to their high yield, and nutritional value. The mechanism employed by the endophytes present in the seed, shoot, leaves, roots, and other microbes inhabiting the rhizosphere, bulk soil in plant growth promotion, and disease control still needs to be emphasized, although some research information are available on them. The variation in the diversity and population of microbes inhabiting different plant parts can be due to difference in the geographical locations, cropping system, developmental stage of the plants and the farming practices adopted. These key factors may affect the crop yield, microbial diversity and their ability to produce secondary metabolites. It is therefore very important to understand the mechanisms behind the production of secondary metabolites in soybean as a measure to improve their production, oil content, antioxidant content, seed quality, carbohydrates, chemical composition, and yield in different environments and also as a model to the research of other crops. More research should also be carried out to help understand the use of endophytes in the agriculture, industry, and medical industries, owing to the production of bioproducts. Better still, there is a need to develop robust bioinformatics tools and analytical techniques with the existing technologies to generate microbiome data as a guide for further experiments. Employing this approach by ecologists, environmental and computational scientists, microbiologists, agriculturists, and industrialists aims to provide insights into plant microbiome research as a reference for further studies. Hence, the authors conclude and recommend that the current approaches highlighted in this review will be of help to researchers in understanding the dynamics, prospect, and potential of endophytic microbes in soybeans and other food crops as agricultural bio-input to ensure food security and sustainable agriculture.

## References

[1] Zhou Y, Zhao W, Lai Y, Zhang B, Zhang D (2020). Edible plant oil: global status, health issues, and perspectives. Front Plant Sci.

[2] Page KL, Dang YP, Martinez C, Dalal RC, Wehr JB, Kopittke PM, Orton TG, Menzies NW (2021). Review of crop-specific tolerance limits to acidity, salinity, and sodicity for seventeen cereal, pulse, and oilseed crops common to rainfed subtropical cropping systems. Land Degrad Develop.

[3] Raipuria RK, Watts A, Meena NL, Watts A (2021) Genome editing in oilseed crops. Genome Editing in Plants, 1st Edition. CRC Press. Page 109–126

[4] Bagnall DK, Shanahan JF, Flanders A, Morgan CL, Honeycutt CW (2021). Soil health considerations for global food security. Agron J.

[5] Paul AA, Kumar S, Kumar V, Sharma R (2020). Milk Analog: plant based alternatives to conventional milk, production, potential and health concerns. Crit Rev Food Sci Nutr.

[6] Velten S, Neumann C, Bleyer M, Gruber-Dujardin E, Hanuszewska M, Przybylska-Gornowicz B, Liebert F (2018). Effects of 50 percent substitution of soybean meal by alternative proteins from *Hermetia illucens* or *Spirulina platensis* in meat-type chicken diets with graded amino acid supply. Open J Animal Sci.

[7] Khojely DM, Ibrahim SE, Sapey E, Han T (2018). History, current status, and prospects of soybean production and research in sub-Saharan Africa. The Crop Journal.

[8] Siamabele B (2021). The significance of soybean production in the face of changing climates in Africa. Cogent Food Agric.

[9] Zhao L, Xu Y, Lai X (2018). Antagonistic endophytic bacteria associated with nodules of soybean (*Glycine max* L.) and plant growth-promoting properties. Braz J Microbiol.

[10] Sallam N, Ali EF, Seleim MA, Bagy HMK (2021). Endophytic fungi associated with soybean plants and their antagonistic activity against *Rhizoctonia solani*. Egypt J Biol Pest Control.

[11] Amrate PK, Shrivastava M, Pancheshwar DK, Sharma S (2020). Charcoal rot and yellow mosaic virus diseases of soybean under hot spot condition: symptoms, incidence and resistance characterization. Int J Biores Stress Manag.

[12] Eid AM, Fouda A, Abdel-Rahman MA, Salem SS, Elsaied A, Oelmüller R, Hijri M, Bhowmik A, Elkelish A, Hassan SE-D (2021). Harnessing bacterial endophytes for promotion of plant growth and biotechnological applications: an overview. Plants.

[13] Akanmu AO, Babalola OO, Venturi V, Ayilara MS, Adeleke BS, Amoo AE, Sobowale AA, Fadiji AE, Glick BR (2021). Plant disease management: leveraging on the plant-microbe-soil interface in the biorational use of organic amendments. Front Plant Sci.

[14] Jordaan E, van der Waals JE, McLaren NW (2019). Effect of irrigation on charcoal rot severity, yield loss and colonization of soybean and sunflower. Crop Prot.

[15] Dubey A, Saiyam D, Kumar A, Hashem A, Abd_Allah EF, Khan ML (2021) Bacterial root endophytes: characterization of their competence and plant growth promotion in soybean (*Glycine max* (L.) Merr.) under drought stress. Int J Environ Res Public Health 18:931 10.3390/ijerph18030931 PMC7908378 33494513

[16] Santoyo G, Moreno-Hagelsieb G, del Carmen O-M, Glick BR (2016). Plant growth-promoting bacterial endophytes. Microbiol Res.

[17] Etesami H (2022). Root nodules of legumes: a suitable ecological niche for isolating non-rhizobial bacteria with biotechnological potential in agriculture. Curr Res Biotechnol.

[18] Bhutani N, Maheshwari R, Kumar P, Suneja P (2021). Bioprospecting of endophytic bacteria from nodules and roots of *Vigna radiata, Vigna unguiculata* and *Cajanus cajan* for their potential use as bioinoculants. Plant Gene.

[19] Mashiane AR, Adeleke RA, Bezuidenhout CC, Chirima GJ (2018). Community composition and functions of endophytic bacteria of Bt maize. South Afr J Sci.

[20] Zhao J, Wang S, Zhu X, Wang Y, Liu X, Duan Y, Fan H, Chen L (2021). Isolation and characterization of nodules endophytic bacteria *Pseudomonas protegens* Sneb 1997 and* Serratia plymuthic*a Sneb2001 for the biological control of root-knot nematode. Appl Soil Ecol.

[21] Bashir S, Iqbal A, Hasnain S, White JF (2021). Screening of sunflower associated bacteria as biocontrol agents for plant growth promotion. Arch Microbiol.

[22] Adeleke BS, Babalola OO (2022). Meta-omics of endophytic microbes in agricultural biotechnology. Biocatal Agric Biotechnol.

[23] Sheteiwy MS, AbdElgawad H, Xiong YC, Macovei A, Brestic M, Skalicky M, Shaghaleh H, Hamoud YA, El-Sawah AM (2021). Inoculation with *Bacillus amyloliquefaciens* and mycorrhiza confers tolerance to drought stress and improve seed yield and quality of soybean plant. Physiol Plantarum.

[24] Wu W, Chen W, Liu S, Wu J, Zhu Y, Qin L, Zhu B (2021). Beneficial relationships between endophytic bacteria and medicinal plants. Front Plant Sci.

[25] Schmidt C, Mrnka L, Lovecká P, Frantík T, Fenclová M, Demnerová K, Vosátka M (2021). Bacterial and fungal endophyte communities in healthy and diseased oilseed rape and their potential for biocontrol of *Sclerotinia* and *Phoma* disease. Sci Rep.

[26] Cui W, He P, Munir S, He P, Li X, Li Y, Wu J, Wu Y, Yang L, He P, He Y (2019). Efficacy of plant growth promoting bacteria Bacillus amyloliquefaciens B9601–Y2 for biocontrol of southern corn leaf blight. Biol Control.

[27] Bashir I, War AF, Rafiq I, Reshi ZA, Rashid I, Shouche YS (2022). Phyllosphere microbiome: diversity and functions. Microbiol Res.

[28] Saber M, Andreote F, Kavamura V, Frighetto R, Taketani R, de MELO I (2014). Effect of ultraviolet-B (UV-B) radiation on bacterial community in the soybean phyllosphere. Afr J Microbiol Res.

[29] Zheng Y, Xu Z, Liu H, Liu Y, Zhou Y, Meng C, Ma S, Xie Z, Li Y, Zhang C-S (2021). Patterns in the microbial community of salt-tolerant plants and the functional genes associated with Salt stress alleviation. Microbiol Spectrum.

[30] Mahgoub HA, Fouda A, Eid AM, Ewais EE-D, Hassan SE-D (2021). Biotechnological application of plant growth-promoting endophytic bacteria isolated from halophytic plants to ameliorate salinity tolerance of *Vicia faba* L. Plant Biotechnol Reports.

[31] Hereira-Pacheco SE, Navarro-Noya YE, Dendooven L (2021). The root endophytic bacterial community of *Ricinus communis* L. resembles the seeds community more than the rhizosphere bacteria independent of soil water content. Sci Rep.

[32] Sugiyama A (2019). The soybean rhizosphere: metabolites, microbes, and beyond - a review. J Adv Res.

[33] Mina D, Pereira JA, Lino-Neto T, Baptista P (2020). Epiphytic and endophytic bacteria on olive tree phyllosphere: exploring tissue and cultivar effect. Microbial Ecol.

[34] de Almeida LK, Carpentieri-Pipolo V, Oro T, Stefani Pagliosa E, Degrassi G (2016). Culturable endophytic bacterial communities associated with field-grown soybean. J Appl Microbiol.

[35] Hwang H-H, Chien P-R, Huang F-C, Hung S-H, Kuo C-H, Deng W-L, Chiang E-PI, Huang C-C (2021). A plant endophytic bacterium, *Burkholderia seminalis* strain 869T2, promotes plant growth in *Arabidopsis*, Pak Choi, Chinese Amaranth, Lettuces, and other vegetables. Microorganisms.

[36] Brunda K, Jahagirdar S, Kambrekar D (2018). Antagonistic activity of bacterial endophytes against major soil borne pathogens of soybean. J Entomol Zool Stud.

[37] Zhang C, Ma X, Zhu R, Liu Z, Gu M, Zhang J, Li Y, Xu Y, Zhu D (2020). Analysis of the endophytic bacteria community structure and function of *Panax notoginseng* based on high-throughput sequencing. Curr Microbiol.

[38] Tangapo AM, Astuti DI, Aditiawati P (2018). Dynamics and diversity of cultivable rhizospheric and endophytic bacteria during the growth stages of cilembu sweet potato (*Ipomoea batatas* L. var. cilembu). Agric Nat Res.

[39] Chlebek D, Pinski A, Żur J, Michalska J, Hupert-Kocurek K (2020). Genome Mining and Evaluation of the biocontrol potential of *Pseudomonas fluorescens* BRZ63, a new endophyte of oilseed rape (*Brassica napus* L.) against fungal pathogens. Int J Mol Sci.

[40] Adeleke BS, Ayangbenro AS, Babalola OO (2021). Genomic analysis of endophytic *Bacillus cereus* T4S and its plant growth-promoting traits. Plants.

[41] Lareen A, Burton F, Schäfer P (2016). Plant root-microbe communication in shaping root microbiomes. Plant Mol Biol.

[42] Feng N-X, Yu J, Zhao H-M, Cheng Y-T, Mo C-H, Cai Q-Y, Li Y-W, Li H, Wong M-H (2017). Efficient phytoremediation of organic contaminants in soils using plant–endophyte partnerships. Sci Total Environ.

[43] Shah S, Shrestha R, Maharjan S, Selosse M-A, Pant B (2019). Isolation and characterization of plant growth-promoting endophytic fungi from the roots of *Dendrobium moniliforme*. Plants.

[44] Adeleke BS, Babalola OO (2021). Biotechnological overview of agriculturally important endophytic fungi. Hortic Environ Biotechnols.

[45] Ripa FA, Cao W-d, Tong S, Sun J-g (2019). Assessment of plant growth promoting and abiotic stress tolerance properties of wheat endophytic fungi. BioMed Res Int.

[46] Kuźniar A, Włodarczyk K, Wolińska A (2019). Agricultural and other biotechnological applications resulting from trophic plant-endophyte interactions. Agron.

[47] Adeleke BS, Babalola OO (2021). The plant endosphere-hidden treasures: a review of fungal endophytes. Biotechnol Genet Eng Rev.

[48] El-Bialy HA, El-Bastawisy HS (2020). Elicitors stimulate paclitaxel production by endophytic fungi isolated from ecologically altered Taxus baccata. J Radiation Res Appl Sci.

[49] Wu Y-Y, Zhang T-Y, Zhang M-Y, Cheng J, Zhang Y-X (2018). An endophytic fungi of *Ginkgo biloba* L. produces antimicrobial metabolites as potential inhibitors of FtsZ of *Staphylococcus aureus*. Fitoterapia.

[50] Hou X, Guo S (2014). Screening and identification of endophytic fungi with growth promoting effect on Dendrobium officinale. China J Chin Mater Med.

[51] Zhang Q, Zhang J, Yang L, Zhang L, Jiang D, Chen W, Li G (2014). Diversity and biocontrol potential of endophytic fungi in *Brassica napus*. Biol Control.

[52] Yu J, Wu Y, He Z, Li M, Zhu K, Gao B (2018). Diversity and antifungal activity of endophytic fungi associated with *Camellia oleifera*. Mycobiol.

[53] Kumar S, Kaushik N (2013). Endophytic fungi isolated from oil-seed crop *Jatropha curcas* produces oil and exhibit antifungal activity. PLoS ONE.

[54] da Costa Stuart AK, Stuart RM, Pimentel IC (2018). Effect of agrochemicals on endophytic fungi community associated with crops of organic and conventional soybean (*Glycine max* L. Merril). Agric Natural Res.

[55] Dos Santos IR, Abdel-Azeem AM, Mohesien MT, Piekutowska M, Sheir DH, da Silva LL, da Silva CC, Carvalho DDC, Bezerra JDP, Saad HA (2021). Insights into the bioprospecting of the endophytic fungi of the medicinal plant *Palicourea rigida* Kunth (Rubiaceae): Detailed biological activities. J Fungi.

[56] Xiao J-l, Sun J-G, Pang B, Zhou X, Gong Y, Jiang L, Zhang L, Ding X, Yin J (2021). Isolation and screening of stress-resistant endophytic fungus strains from wild and cultivated soybeans in cold region of China. Appl Microbiol Biotechnol.

[57] Ancheeva E, Daletos G, Proksch P (2020). Bioactive secondary metabolites from endophytic fungi. Curr Med Chem.

[58] Skiada V, Faccio A, Kavroulakis N, Genre A, Bonfante P, Papadopoulou KK (2019). Colonization of legumes by an endophytic *Fusarium solani* strain FsK reveals common features to symbionts or pathogens. Fungal Genet Biol.

[59] Munshi M, Sohrab M, Begum M, Rony SR, Karim M, Afroz F, Hasan M (2021). Evaluation of bioactivity and phytochemical screening of endophytic fungi isolated from *Ceriops decandra* (Griff.) W. Theob, a mangrove plant in Bangladesh. Clin Phytosci.

[60] Thomas P, Swarna GK, Roy PK, Patil P (2008). Identification of culturable and originally non-culturable endophytic bacteria isolated from shoot tip cultures of banana cv. Grand Naine. Plant Cell Tissue Organ Culture.

[61] Adeleke BS, Ayangbenro AS, Babalola OO (2021). Bacterial community structure of the sunflower (*Helianthus annuus*) endosphere. Plant Signaling Behav.

[62] Bolivar-Anillo HJ, González-Rodríguez VE, Cantoral JM, García-Sánchez D, Collado IG, Garrido C (2021). Endophytic bacteria *Bacillus subtilis*, isolated from *Zea mays*, as potential biocontrol agent against *Botrytis cinerea*. Biol.

[63] Afzal I, Shinwari ZK, Sikandar S, Shahzad S (2019). Plant beneficial endophytic bacteria: mechanisms, diversity, host range and genetic determinants. Microbiol Res.

[64] Alain K, Querellou J (2009). Cultivating the uncultured: limits, advances and future challenges. Extremophiles.

[65] Torsvik V, Ovresas L (2002). Microbial diversity and function in soil: from genes to ecosystems. Curr Opin Microbiol.

[66] Garcias-Bonet N, Arrieta JM, de Santana CN, Duarte CM, Marbà N (2012). Endophytic bacterial community of a Mediterranean marine angiosperm (*Posidonia oceanica*). Front Microbiol.

[67] Piccolo SL, Ferraro V, Alfonzo A, Settanni L, Ercolini D, Burruano S, Moschetti G (2010). Presence of endophytic bacteria in *Vitis vinifera* leaves as detected by fluorescence in situ hybridization. Ann Microbiol.

[68] Ikeda S, Kaneko T, Okubo T, Rallos LE, Eda S, Mitsui H, Sato S, Nakamura Y, Tabata S, Minamisawa K (2009). Development of a bacterial cell enrichment method and its application to the community analysis in soybean stems. Microbial Ecol.

[69] Lundberg DS, Yourstone S, Mieczkowski P, Jones CD, Dangl JL (2013). Practical innovations for high-throughput amplicon sequencing. Nat Methods.

[70] Kaul S, Sharma T, Dhar K, M,  (2016). “Omics” tools for better understanding the plant–endophyte interactions. Front Plant Sci.

[71] Adeleke BS, Babalola OO, Glick BR (2021). Plant growth-promoting root-colonizing bacterial endophytes. Rhizosph.

[72] Dos Santos LF, Olivares FL (2021). Plant microbiome structure and benefits for sustainable agriculture. Curr Plant Biol.

[73] Hartman K, van der Heijden MG, Roussely-Provent V, Walser J-C, Schlaeppi K (2017). Deciphering composition and function of the root microbiome of a legume plant. Microbiome.

[74] Eldridge DJ, Travers SK, Val J, Ding J, Wang JT, Singh BK, Delgado-Baquerizo M (2021). Experimental evidence of strong relationships between soil microbial communities and plant germination. J Ecol.

[75] Mishra VK, Passari AK, Leo VV, Singh BP (2017) Molecular diversity and detection of endophytic fungi based on their antimicrobial biosynthetic genes. In: Singh BP, Gupta VK (eds) Molecular markers in mycology. Fungal biology. Springer, Cham, pp 1–35. 10.1007/978-3-319-34106-4_1

[76] Mitter B, Pfaffenbichler N, Flavell R, Compant S, Antonielli L, Petric A, Berninger T, Naveed M, Sheibani-Tezerji R, von Maltzahn G (2017). A new approach to modify plant microbiomes and traits by introducing beneficial bacteria at flowering into progeny seeds. Front Microbiol.

[77] Bodenhausen N, Bortfeld-Miller M, Ackermann M, Vorholt JA (2014). A synthetic community approach reveals plant genotypes affecting the phyllosphere microbiota. PLoS Genet.

[78] Dubey RK, Tripathi V, Prabha R, Chaurasia R, Singh DP, Rao CS, El-Keblawy A, Abhilash PC (2020) Methods for exploring soil microbial diversity. In: Dubey RK, Tripathi V, Prabha R, Chaurasia R, Singh DP, Rao CS, El-Keblawy A, Abhilash PC (eds) Unravelling the soil microbiome: perspectives for environmental sustainability. Springer International Publishing, pp 23–32. 10.1007/978-3-030-15516-2_3

[79] İnceoğlu Ö, Salles JF, van Overbeek L, van Elsas JD (2010). Effects of plant genotype and growth stage on the betaproteobacterial communities associated with different potato cultivars in two fields. Appl Environ Microbiol.

[80] Hayatsu M, Tago K, Saito M (2008). Various players in the nitrogen cycle: diversity and functions of the microorganisms involved in nitrification and denitrification. Soil Sci Plant Nutr.

[81] Qiu P, Feng Z-X, Tian J-W, Lei Z-C, Wang L, Zeng Z-G, Chu Y-W, Tian Y-Q (2015). Diversity, bioactivities, and metabolic potentials of endophytic actinomycetes isolated from traditional medicinal plants in Sichuan, China. Chin J Nat Med.

[82] Allan E (2014). Metagenomics: unrestricted access to microbial communities. Virulence.

[83] Shahzad R, Khan AL, Bilal S, Waqas M, Kang S-M, Lee I-J (2017). Inoculation of abscisic acid-producing endophytic bacteria enhances salinity stress tolerance in *Oryza** sativa*. Environ Exp Bot.

[84] Pei C, Mi C, Sun L, Liu W, Li O, Hu X (2017). Diversity of endophytic bacteria of *Dendrobium officinale* based on culture-dependent and culture-independent methods. Biotechnol Biotechnolog Equip.

[85] Gupta R, Anand G, Gaur R, Yadav D (2021). Plant–microbiome interactions for sustainable agriculture: a review. Physiol Mol Biol Plants.

[86] Aswani R, Thomas R, Radhakrishnan EK (2022) Induction of plant defense response by endophytic microorganisms. In: Radhakrishnan EK, Ajay K, Aswani R (eds) Biocontrol mechanisms of endophytic microorganisms. Academic Press, Cambridge, pp 89–115. 10.1016/B978-0-323-88478-5.00002-X

[87] Samreen T, Naveed M, Nazir MZ, Asghar HN, Khan MI, Zahir ZA, Kanwal S, Jeevan B, Sharma D, Meena VS (2021). Seed associated bacterial and fungal endophytes: diversity, life cycle, transmission, and application potential. Appl Soil Ecol.

[88] Gaeth VA, Domondon CJ, Podbielski PA, Aswad VX, Wrightstone EA, Wong NH, Burke WH, Melita J, Murray KM, Hudson AO (2021). Whole-genome sequencing and annotation of 10 endophytic and epiphytic bacteria isolated from *Lolium arundinaceum*. Microbiol Res Announc.

[89] Samaras A, Nikolaidis M, Antequera-Gómez ML, Cámara-Almirón J, Romero D, Moschakis T, Amoutzias GD, Karaoglanidis GS (2020). Whole genome sequencing and root colonization studies reveal novel insights in the biocontrol potential and growth promotion by *Bacillus subtilis* MBI 600 on cucumber. Front Microbiol.

[90] Adeleke BS, Ayangbenro AS, Babalola OO (2021). Genomic assessment of *Stenotrophomonas indicatrix* for improved sunflower plant. Curr Genet.

[91] Ulrich K, Kube M, Becker R, Schneck V, Ulrich A (2021). Genomic analysis of the endophytic *Stenotrophomonas* strain 169 reveals features related to plant-growth promotion and stress tolerance. Front Microbiol.

[92] Kaewkla O, Franco CMM (2021). *Amycolatopsis pittospori* sp. nov., an endophytic actinobacterium isolated from native apricot tree and genome mining revealed the biosynthesis potential as antibiotic producer and plant growth promoter. Antonie Van Leeuwenhoek.

[93] Ku Y, Xu G, Tian X, Xie H, Yang X, Cao C (2018). Root colonization and growth promotion of soybean, wheat and Chinese cabbage by *Bacillus cereus* YL6. PLoS ONE.

[94] Kumawat KC, Singh I, Napgal S, Sharma P, Gupta RK, Sirari A (2022). Co-inoculation of indigenous *Pseudomonas oryzihabitans* and *Bradyrhizobium* sp. modulates the growth, symbiotic efficacy, nutrient acquisition, and grain yield of soybean. Pedosph.

[95] Nwachukwu BC, Ayangbenro AS, Babalola OO (2021). Elucidating the rhizosphere associated bacteria for environmental sustainability. Agric.

[96] Babalola OO, Emmanuel OC, Adeleke BS, Odelade KA, Nwachukwu BC, Ayiti OE, Adegboyega TT, Igiehon NO (2021). Rhizosphere microbiome cooperations: strategies for sustainable crop production. Curr Microbiol.

[97] Adedeji AA, Babalola OO (2020). Secondary metabolites as plant defensive strategy: a large role for small molecules in the near root region. Planta.

[98] Chen L, Brookes PC, Xu J, Zhang J, Zhang C, Zhou X, Luo Y (2016). Structural and functional differentiation of the root-associated bacterial microbiomes of perennial ryegrass. Soil Biol Biochem.

[99] Wongdee J, Yuttavanichakul W, Longthonglang A, Teamtisong K, Boonkerd N, Teaumroong N, Tittabutr P (2021) Enhancing the efficiency of soybean inoculant for nodulation under multi-environmental stress conditions. Polish J Microbiol 70:257 10.33073/pjm-2021-024 PMC8326982 34349815

[100] Bavaresco LG, Osco LP, Araujo ASF, Mendes LW, Bonifacio A, Araujo FF (2020). *Bacillus subtilis* can modulate the growth and root architecture in soybean through volatile organic compounds. Theor Exp Plant Physiol.

[101] Sibponkrung S, Kondo T, Tanaka K, Tittabutr P, Boonkerd N, Teaumroong N, Yoshida K-i (2017). Genome sequence of *Bacillus velezensis* S141, a new strain of plant growth-promoting rhizobacterium isolated from soybean rhizosphere. Microbiol Res Announc.

[102] Preece C, Penuelas J (2016). Rhizodeposition under drought and consequences for soil communities and ecosystem resilience. Plant Soil.

[103] Schlatter DC, Hansen JC, Schillinger WF, Sullivan TS, Paulitz TC (2019). Common and unique rhizosphere microbial communities of wheat and canola in a semiarid Mediterranean environment. Appl Soil Ecol.

[104] Qiao Q, Wang F, Zhang J, Chen Y, Zhang C, Liu G, Zhang H, Ma C, Zhang J (2017). The variation in the rhizosphere microbiome of cotton with soil type, genotype and developmental stage. Sci Rep.

[105] Xing M, Zhang Y, Guan C, Guan M (2021) Effects of nitrogen application rate on rhizosphere microbial diversity in oilseed Rape (*Brassica napus* L.). Agron 11:1539

[106] Liu Y, Gao J, Bai Z, Wu S, Li X, Wang N, Du X, Fan H, Zhuang G, Bohu T (2021) Unraveling mechanisms and impact of microbial recruitment on oilseed rape (*Brassica napus* L.) and the rhizosphere mediated by plant growth-promoting rhizobacteria. Microorganisms 9:161 10.3390/microorganisms9010161 PMC7828142 33445684

[107] Sohn S-I, Ahn J-H, Pandian S, Oh Y-J, Shin E-K, Kang H-J, Cho W-S, Cho Y-S, Shin K-S (2021). Dynamics of bacterial community structure in the rhizosphere and root nodule of soybean: Impacts of growth stages and varieties. Int J Mol Sci.

[108] Cordero Elvia J, de Freitas RJ, Germida J (2021). Bacterial microbiome associated with the rhizosphere, root interior and aboveground plant organs of wheat and canola at different growth stages. Phytobiomes J.

[109] Mavrodi OV, McWilliams JR, Peter JO, Berim A, Hassan KA, Elbourne LD, LeTourneau MK, Gang DR, Paulsen IT, Weller DM (2021). Root exudates alter the expression of diverse metabolic, transport, regulatory, and stress response genes in rhizosphere *Pseudomonas*. Front Microbiol.

[110] Soleymani A (2019). Safflower (Carthamus tinctorius L.) seed vigor tests for the prediction of field emergence. Ind Crops Products.

[111] Vandana U, Rajkumari J, Singha L, Satish L, Alavilli H, Sudheer P, Chauhan S, Ratnala R, Satturu V, Mazumder P (2021). The endophytic microbiome as a hotspot of synergistic interactions, with prospects of plant growth promotion. Biol.

[112] Rahman MDM, Flory E, Koyro H-W, Abideen Z, Schikora A, Suarez C, Schnell S, Cardinale M (2018). Consistent associations with beneficial bacteria in the seed endosphere of barley (*Hordeum vulgare* L.). Syst Appl Microbiol.

[113] Ghorbanpour M, Hatami M (2014). Biopriming of salvia officinalis seed with growth promoting rhizobacteria affects invigoration and germination indices. J Biol Environ Sci.

[114] Omomowo OI, Babalola OO (2019). Bacterial and fungal endophytes: tiny giants with immense beneficial potential for plant growth and sustainable agricultural productivity. Microorganisms.

[115] Khan AL, Gilani SA, Waqas M, Al-Hosni K, Al-Khiziri S, Kim Y-H, Ali L, Kang S-M, Asaf S, Shahzad R (2017). Endophytes from medicinal plants and their potential for producing indole acetic acid, improving seed germination and mitigating oxidative stress. J Zhejiang University-Sci B.

[116] Abdelfattah A, Wisniewski M, Schena L, Tack AJ (2021). Experimental evidence of microbial inheritance in plants and transmission routes from seed to phyllosphere and root. Environ Microbiol.

[117] Lamichhane JR, Debaeke P, Steinberg C, You MP, Barbetti MJ, Aubertot J-N (2018). Abiotic and biotic factors affecting crop seed germination and seedling emergence: a conceptual framework. Plant Soil.

[118] Dhole A, Shelat H, Vyas R, Jhala Y, Bhange M (2016). Endophytic occupation of legume root nodules by nifH-positive non-rhizobial bacteria, and their efficacy in the groundnut (*Arachis hypogaea*). Annals Microbiol.

[119] Sánchez-López AS, Thijs S, Beckers B, González-Chávez MC, Weyens N, Carrillo-González R, Vangronsveld J (2018). Community structure and diversity of endophytic bacteria in seeds of three consecutive generations of *Crotalaria pumila* growing on metal mine residues. Plant Soil.

[120] Mukherjee A, Gaurav AK, Patel AK, Singh S, Chouhan GK, Lepcha A, Pereira APdA, Verma JP (2021). Unlocking the potential plant growth-promoting properties of chickpea (*Cicer arietinum* L.) seed endophytes bio-inoculants for improving soil health and crop production. Land Degradation Develop.

[121] Kelly C, Haddix M, Byrne P, Cotrufo MF, Schipanski M, Kallenbach C, Wallenstein M, Fonte SJ (2021). Divergent belowground carbon allocation patterns of winter wheat shape rhizosphere microbial communities and nitrogen cycling activities. Soil Biol Biochem.

[122] Nascimento FX, Hernández AG, Glick BR, Rossi MJ (2020). Plant growth-promoting activities and genomic analysis of the stress-resistant *Bacillus megaterium* STB1, a bacterium of agricultural and biotechnological interest. Biotechnol Rep.

[123] Rana KL, Kour D, Kaur T, Sheikh I, Yadav AN, Kumar V, Suman A, Dhaliwal HS (2020). Endophytic microbes from diverse wheat genotypes and their potential biotechnological applications in plant growth promotion and nutrient uptake. Proceed National Acad Sci.

[124] Krishnamoorthy A, Gupta A, Sar P, Maiti MK (2021). Metagenomics of two gnotobiotically grown aromatic rice cultivars reveals genotype-dependent and tissue-specific colonization of endophytic bacterial communities attributing multiple plant growth promoting traits. World J Microbiol Biotechnol.

[125] Jie W-G, Yao Y-X, Guo N, Zhang Y-Z, Qiao W (2021). Effects of *Rhizophagus intraradices* on plant growth and the composition of microbial communities in the roots of continuous cropping soybean at maturity. Sustainability.

[126] Van Bruggen A, Finckh M (2016). Plant diseases and management approaches in organic farming systems. Ann Rev Phytopathol.

[127] Tyagi J, Chaudhary P, Mishra A, Khatwani M, Dey S, Varma A (2022). Role of endophytes in abiotic stress tolerance: with special emphasis on *Serendipita indica*. Int J Environ Res.

[128] Orozco-Mosqueda M, Flores A, Rojas-Sánchez B, Urtis-Flores CA, Morales-Cedeño LR, Valencia-Marin MF, Chávez-Avila S, Rojas-Solis D, Santoyo G (2021). Plant growth-promoting bacteria as bioinoculants: attributes and challenges for sustainable crop improvement. Agron.

[129] Müller CA, Obermeier MM, Berg G (2016). Bioprospecting plant-associated microbiomes. J Biotechnol.

[130] Mahanty T, Bhattacharjee S, Goswami M, Bhattacharyya P, Das B, Ghosh A, Tribedi P (2017). Biofertilizers: a potential approach for sustainable agriculture development. Environ Sci Poll Res.

[131] Fadiji AE, Babalola OO (2020). Elucidating mechanisms of endophytes used in plant protection and other bioactivities with multifunctional prospects. Front Bioeng Biotechnol.

[132] Latz MA, Jensen B, Collinge DB, Jørgensen HJ (2018). Endophytic fungi as biocontrol agents: elucidating mechanisms in disease suppression. Plant Ecol Diversity.

[133] Rojas EC, Jensen B, Jørgensen HJ, Latz MA, Esteban P, Ding Y, Collinge DB (2020). Selection of fungal endophytes with biocontrol potential against *Fusarium* head blight in wheat. Biol Control.

[134] Marag PS, Suman A, Gond S (2018). Prospecting endophytic bacterial colonization and their potential plant growth promoting attributes in hybrid maize (*Zea mays* L.). Int J Curr Microbiol Appl Sci.

[135] Kumawat K, Sharma P, Sirari A, Singh I, Gill B, Singh U, Saharan K (2019). Synergism of *Pseudomonas aeruginosa* (LSE-2) nodule endophyte with *Bradyrhizobium* sp. (LSBR-3) for improving plant growth, nutrient acquisition and soil health in soybean. World J Microbiol Biotechnol.

[136] Liu Y-Q, Wang Y-H, Kong W-L, Liu W-H, Xie X-L, Wu X-Q (2020). Identification, cloning and expression patterns of the genes related to phosphate solubilization in *Burkholderia multivorans* WS-FJ9 under different soluble phosphate levels. AMB Express.

[137] Behera BC, Singdevsachan SK, Mishra RR, Dutta SK, Thatoi HN (2014). Diversity, mechanism and biotechnology of phosphate solubilising microorganism in mangrove - a review. Biocatal Agric Biotechnol.

[138] Valetti L, Iriarte L, Fabra A (2018). Growth promotion of rapeseed (*Brassica napus*) associated with the inoculation of phosphate solubilizing bacteria. Appl Soil Ecol.

[139] Shahid M, Hameed S, Tariq M, Zafar M, Ali A, Ahmad N (2015). Characterization of mineral phosphate-solubilizing bacteria for enhanced sunflower growth and yield-attributing traits. Annals Microbiol.

[140] Lucero CT, Lorda GS, Ludueña LM, Anzuay MS, Taurian T (2020). Motility and biofilm production involved in the interaction of phosphate solubilizing endophytic strains with peanut, maize and soybean plants. Rhizosph.

[141] Kenasa G, Nandeshwar B, Assefa F (2021). *In vitro* inorganic phosphate solubilization tests of cowpea root nodule bacteria from Ethiopia. Agric Sci Digest.

[142] Yasmeen S, Bano A (2014). Combined effect of phosphate-solubilizing microorganisms, *Rhizobium* and *Enterobacter* on root nodulation and physiology of soybean (*Glycine max* L.). Comm Soil Sci Plant Analysis.

[143] Tariq M, Hameed S, Yasmeen T, Zahid M, Zafar M (2014). Molecular characterization and identification of plant growth promoting endophytic bacteria isolated from the root nodules of pea (*Pisum sativum* L.). World J Microbiol Biotechnol.

[144] Sánchez-Cruz R, Tpia Vázquez I, Batista-García RA, Méndez-Santiago EW, Sánchez-Carbente MdR, Leija A, Lira-Ruan V, Hernández G, Wong-Villarreal A, Folch-Mallol JL (2019). Isolation and characterization of endophytes from nodules of Mimosa pudica with biotechnological potential. Microbiol Res.

[145] Chaudhary A, Chaudhary P, Upadhyay A, Kumar A, Singh A (2021). Effect of *Gypsum* on plant growth promoting rhizobacteria. Environ Ecol.

[146] Bertalan M, Albano R, de Pádua V, Rouws L, Rojas C, Hemerly A, Teixeira K, Schwab S, Araujo J, Oliveira A (2009). Complete genome sequence of the sugarcane nitrogen-fixing endophyte *Gluconacetobacter diazotrophicus* Pal5. BMC Genomics.

[147] El Attar I, Taha K, El Bekkay B, El Khadir M, Thami Alami I, Aurag J (2019). Screening of stress tolerant bacterial strains possessing interesting multi-plant growth promoting traits isolated from root nodules of Phaseolus vulgaris L. Biocatal Agric Biotechnol.

[148] Ferreira CMH, Soares HMVM, Soares EV (2019). Promising bacterial genera for agricultural practices: an insight on plant growth-promoting properties and microbial safety aspects. Sci Total Environ.

[149] Maheshwari R, Bhutani N, Suneja P (2019). Screening and characterization of siderophore producing endophytic bacteria from *Cicer arietinum* and *Pisum sativum* plants. J Applied Biol Biotechnol.

[150] Zaman NR, Chowdhury UF, Reza RN, Chowdhury FT, Sarker M, Hossain MM, Akbor MA, Amin A, Islam MR, Khan H (2021). Plant growth promoting endophyte *Burkholderia contaminans* NZ antagonizes phytopathogen *Macrophomina phaseolina* through melanin synthesis and pyrrolnitrin inhibition. PLoS ONE.

[151] Gao J-l, Sun P, Sun Y-c, Xue J, Wang G, Wang L-w, Du Y, Zhang X, Sun J-g (2021). *Caulobacter* endophyticus sp. nov., an endophytic bacterium harboring three lasso peptide biosynthetic gene clusters and producing indoleacetic acid isolated from maize root. Antonie Van Leeuwenhoek.

[152] Ahmad E, Sharma SK, Sharma PK (2020) Deciphering operation of tryptophan-independent pathway in high indole-3-acetic acid (IAA) producing *Micrococcus aloeverae* DCB-20. FEMS Microbiol Lett 367:fnaa190 10.1093/femsle/fnaa190 33201985

[153] Pérez-Montaño F, Alías-Villegas C, Bellogín RA, del Cerro P, Espuny MR, Jiménez-Guerrero I, López-Baena FJ, Ollero FJ, Cubo T (2014). Plant growth promotion in cereal and leguminous agricultural important plants: from microorganism capacities to crop production. Microbiol Res.

[154] Sofy MR, Aboseidah AA, Heneidak SA, Ahmed HR (2021). ACC deaminase containing endophytic bacteria ameliorate salt stress in *Pisum sativum* through reduced oxidative damage and induction of antioxidative defense systems. Environ Sci Poll Res.

[155] Montejano-Ramírez V, García-Pineda E, Valencia-Cantero E (2020). Bacterial compound N, N-dimethylhexadecylamine modulates expression of iron deficiency and defense response genes in *Medicago truncatula* independently of the jasmonic acid pathway. Plants.

[156] Conti R, Chagas FO, Caraballo‐Rodriguez AM, Melo WGdP, do Nascimento AM, Cavalcanti BC, de Moraes MO, Pessoa C, Costa‐Lotufo LV, Krogh R (2016) Endophytic actinobacteria from the Brazilian medicinal plant *Lychnophora ericoides* Mart. and the biological potential of their secondary metabolites. Chem Biodiversity 13:727-736 10.1002/cbdv.201500225 27128202

[157] Adeleke BS, Ayangbenro A, Babalola OO (2022). Effect of endophytic bacterium, *Stenotrophomonas maltophilia* JVB5 on sunflowers. Plant Protection Sci.

[158] Fadiji AE, Ayangbenro AS, Babalola OO (2020). Metagenomic profiling of the community structure, diversity, and nutrient pathways of bacterial endophytes in maize plant. Antonie Van Leeuwenhoek.

[159] Maropola MKA, Ramond J-B, Trindade M (2015). Impact of metagenomic DNA extraction procedures on the identifiable endophytic bacterial diversity in *Sorghum bicolor* (L. Moench). J Microbiol Methods.

[160] Bullington LS, Lekberg Y, Larkin BG (2021). Insufficient sampling constrains our characterization of plant microbiomes. Sci Rep.

[161] Lipková N, Medo J, Artimová R, Maková J, Petrová J, Javoreková S, Michalko J (2021). Growth promotion of rapeseed (*Brassica napus* L.) and blackleg disease (*Leptosphaeria maculans*) suppression mediated by endophytic bacteria. Agron.

[162] Ribeiro IDA, Bach E, da Silva Moreira F, Müller AR, Rangel CP, Wilhelm CM, Barth AL, Passaglia LMP (2021) Antifungal potential against *Sclerotinia sclerotiorum* (Lib.) de Bary and plant growth promoting abilities of *Bacillus* isolates from canola (*Brassica napus* L.) roots. Microbiol Res 248:126754 10.1016/j.micres.2021.126754 33848783

[163] Martínez-Hidalgo P, Flores-Félix JD, Sánchez-Juanes F, Rivas R, Mateos PF, Santa Regina I, Peix Á, Martínez-Molina E, Igual JM, Velázquez E (2021). Identification of *Canola* roots endophytic bacteria and analysis of their potential as biofertilizers for *Canola* crops with special emphasis on sporulating bacteria. Agron.

[164] Jiménez-Gómez A, Saati-Santamaría Z, Kostovcik M, Rivas R, Velázquez E, Mateos PF, Menéndez E, García-Fraile P (2020) Selection of the root endophyte *Pseudomonas brassicacearum* CDVBN10 as plant growth promoter for *Brassica napus* L. crops. Agron 10:1788

[165] Cheng Z, Park E, Glick BR (2007). 1-Aminocyclopropane-1-carboxylate deaminase from *Pseudomonas putida* UW4 facilitates the growth of canola in the presence of salt. Canad J Microbiol.

[166] Trivedi G, Patel P, Saraf M (2020). Synergistic effect of endophytic selenobacteria on biofortification and growth of *Glycine max* under drought stress. South Afr J Bot.

[167] Egamberdieva D, Jabborova D, Berg G (2016). Synergistic interactions between *Bradyrhizobium japonicum* and the endophyte *Stenotrophomonas rhizophila* and their effects on growth, and nodulation of soybean under salt stress. Plant Soil.

[168] Subramanian P, Kim K, Krishnamoorthy R, Sundaram S, Sa T (2015). Endophytic bacteria improve nodule function and plant nitrogen in soybean on co-inoculation with *Bradyrhizobium japonicum* MN110. Plant Growth Reg.

[169] Kim M-J, Radhakrishnan R, Kang S-M, You Y-H, Jeong E-J, Kim J-G, Lee I-J (2017). Plant growth promoting effect of *Bacillus amyloliquefaciens* H-2-5 on crop plants and influence on physiological changes in soybean under soil salinity. Physiol Mol Biol Plants.

[170] Archana T, Rajendran L, Manoranjitham S, Krishnan VS, Paramasivan M, Karthikeyan G (2020) Culture-dependent analysis of seed bacterial endophyte, *Pseudomonas* spp. EGN 1 against the stem rot disease (*Sclerotium rolfsii* Sacc.) in groundnut. Egypt J Biol Pest Control 30:119

[171] Puri A, Padda KP, Chanway CP (2016). Evidence of nitrogen fixation and growth promotion in canola (*Brassica napus* L.) by an endophytic diazotroph *Paenibacillus polymyxa* P2b–2R. Biol Fertility Soils.

[172] Ghavami N, Alikhani HA, Pourbabaei AA, Besharati H (2017). Effects of two new siderophore-producing rhizobacteria on growth and iron content of maize and canola plants. J Plant Nutr.

[173] Li L, Zhang Z, Pan S, Li L, Li X (2019). Characterization and metabolism effect of seed endophytic bacteria associated with peanut grown in south China. Front Microbiol.

[174] Chen L, Shi H, Heng J, Wang D, Bian K (2019). Antimicrobial, plant growth-promoting and genomic properties of the peanut endophyte *Bacillus velezensis* LDO2. Microbiol Res.

[175] Preyanga R, Anandham R, Krishnamoorthy R, Senthilkumar M, Gopal N, Vellaikumar A, Meena S (2021). Groundnut (*Arachis hypogaea*) nodule *Rhizobium* and passenger endophytic bacterial cultivable diversity and their impact on plant growth promotion. Rhizosph.

[176] Wang X, Liang G (2014). Control efficacy of an endophytic *Bacillus amyloliquefaciens* strain BZ6-1 against peanut bacterial wilt, *Ralstonia*
*solanacearum*. BioMed Res Int.

[177] Lucero CT, Lorda GS, Anzuay MS, Ludueña LM, Taurian T (2021). Peanut endophytic phosphate solubilizing bacteria increase growth and P content of soybean and maize plants. Curr Microbiol.

[178] Kumar A, Voropaeva O, Maleva M, Panikovskaya K, Borisova G, Rajkumar M, Bruno LB (2021). Bioaugmentation with copper tolerant endophyte *Pseudomonas lurida* strain EOO26 for improved plant growth and copper phytoremediation by *Helianthus annuus*. Chemosph.

[179] Qadir M, Hussain A, Hamayun M, Shah M, Iqbal A, Irshad M, Ahmad A, Lodhi MA, Lee I-J (2021). Phytohormones producing *Acinetobacter bouvetii* P1 mitigates chromate stress in sunflower by provoking host antioxidant response. Antioxidants.

[180] Selim HM, Gomaa NM, Essa AM (2017). Application of endophytic bacteria for the biocontrol of *Rhizoctonia solani* (Cantharellales: ceratobasidiaceae) damping-off disease in cotton seedlings. Biocontrol Sci Technol.

[181] Yang P, Sun Z-x, Liu S-y, Lu H-x, Zhou Y, Sun M (2013). Combining antagonistic endophytic bacteria in different growth stages of cotton for control of *Verticillium* wilt. Crop Prot.

[182] Pawlik M, Płociniczak T, Thijs S, Pintelon I, Vangronsveld J, Piotrowska-Seget Z (2020). Comparison of two inoculation methods of endophytic bacteria to enhance phytodegradation efficacy of an aged petroleum hydrocarbons polluted soil. Agron.

[183] Wang Q, Ma L, Zhou Q, Chen B, Zhang X, Wu Y, Pan F, Huang L, Yang X, Feng Y (2019). Inoculation of plant growth promoting bacteria from hyperaccumulator facilitated non-host root development and provided promising agents for elevated phytoremediation efficiency. Chemosph.

[184] Diaz PAE, Baron NC, Rigobelo EC (2019). *Bacillus* spp. as plant growth-promoting bacteria in cotton under greenhouse conditions. Austr J Crop Sci.

[185] Rana KL, Kour D, Kaur T, Devi R, Yadav A, Yadav AN (2021). Bioprospecting of endophytic bacteria from the Indian Himalayas and their role in plant growth promotion of maize (*Zea mays* L.). J Appl Biol Biotechnol.

[186] Pal G, Kumar K, Verma A, Verma SK (2022). Seed inhabiting bacterial endophytes of maize promote seedling establishment and provide protection against fungal disease. Microbiol Res.

[187] Naveed M, Mitter B, Reichenauer TG, Wieczorek K, Sessitsch A (2014). Increased drought stress resilience of maize through endophytic colonization by *Burkholderia phytofirmans* PsJN and *Enterobacter* sp. FD17. Environ Exp Bot.

[188] Khan M, Asaf S, Khan A, Adhikari A, Jan R, Ali S, Imran M, Kim KM, Lee IJ (2020). Plant growth-promoting endophytic bacteria augment growth and salinity tolerance in rice plants. Plant Biol.

[189] Lu L, Chang M, Han X, Wang Q, Wang J, Yang H, Guan Q, Dai S (2021). Beneficial effects of endophytic *Pantoea ananatis* with ability to promote rice growth under saline stress. J Appl Microbiol.

[190] Hossain MT, Khan A, Chung EJ, Rashid MH-O, Chung YR (2016). Biological control of rice bakanae by an endophytic *Bacillus oryzicola* YC7007. The Plant Pathol J.

[191] Pan D, Mionetto A, Tiscornia S, Bettucci L (2015). Endophytic bacteria from wheat grain as biocontrol agents of *Fusarium graminearum* and deoxynivalenol production in wheat. Mycotox Res.

[192] Shah D, Khan MS, Aziz S, Ali H, Pecoraro L (2022). Molecular and biochemical characterization, antimicrobial activity, stress tolerance, and plant growth-promoting effect of endophytic bacteria isolated from wheat varieties. Microorganisms.

[193] Govindasamy V, George P, Kumar M, Aher L, Raina SK, Rane J, Annapurna K, Minhas PS (2020) Multi-trait PGP rhizobacterial endophytes alleviate drought stress in a senescent genotype of sorghum [*Sorghum bicolor* (L.) Moench]. 3 Biotech 10:13 10.1007/s13205-019-2001-4 PMC6904756 31879577

[194] Egamberdieva D, Wirth SJ, Shurigin VV, Hashem A, Abd_Allah EF (2017) Endophytic bacteria improve plant growth, symbiotic performance of chickpea (*Cicer arietinum* L.) and induce suppression of root rot caused by *Fusarium solani* under salt stress. Front Microbiol 8:1887 10.3389/fmicb.2017.01887 PMC5625113 29033922

[195] Liu Y, Yan H, Zhang X, Zhang R, Li M, Xu T, Yang F, Zheng H, Zhao J (2020) Investigating the endophytic bacterial diversity and community structures in seeds of genetically related maize (*Zea mays* L.) genotypes. Biotechnol 10:27 10.1007/s13205-019-2034-8 PMC6942555 31950006

[196] Kuramae EE, Derksen S, Schlemper TR, Dimitrov MR, Costa OY, da Silveira AP (2020). Sorghum growth promotion by *Paraburkholderia tropica* and *Herbaspirillum frisingense*: Putative mechanisms revealed by genomics and metagenomics. Microorganisms.

[197] Tian B-Y, Cao Y, Zhang K-Q (2015). Metagenomic insights into communities, functions of endophytes and their associates with infection by root-knot nematode, *Meloidogyne incognita*, in tomato roots. Sci Rep.

[198] Sessitsch A, Hardoim P, Döring J, Weilharter A, Krause A, Woyke T, Mitter B, Hauberg-Lotte L, Friedrich F, Rahalkar M (2012). Functional characteristics of an endophyte community colonizing rice roots as revealed by metagenomic analysis. Mol Plant-Microbe Interact.

[199] Ali M, Ali Q, Sohail MA, Ashraf MF, Saleem MH, Hussain S, Zhou L (2021). Diversity and taxonomic distribution of endophytic bacterial community in the rice plant and its prospective. Int J Mol Sci.

[200] Yang R, Liu P, Ye W (2017). Illumina-based analysis of endophytic bacterial diversity of tree peony (*Paeonia* Sect. Moutan) roots and leaves. Braz J Microbiol.

[201] Hakim S, Mirza BS, Imran A, Zaheer A, Yasmin S, Mubeen F, Mclean JE, Mirza MS (2020) Illumina sequencing of 16S rRNA tag shows disparity in rhizobial and non-rhizobial diversity associated with root nodules of mung bean (*Vigna radiata* L.) growing in different habitats in Pakistan. Microbiol Res 231:126356 10.1016/j.micres.2019.126356 31722286

[202] Brígido C, Singh S, Menéndez E, Tavares MJ, Glick BR, Félix MdR, Oliveira S, Carvalho M (2019). Diversity and functionality of culturable endophytic bacterial communities in chickpea plants. Plants.

[203] Li Y, Wang M, Chen S (2021). Application of N_2_-fixing *Paenibacillus triticisoli* BJ-18 changes the compositions and functions of the bacterial, diazotrophic, and fungal microbiomes in the rhizosphere and root/shoot endosphere of wheat under field conditions. Biol Fertility Soils.

[204] Iqrar I, Numan M, Khan T, Shinwari ZK, Ali GS (2021). LC–MS/MS-based profiling of bioactive metabolites of endophytic bacteria from Cannabis sativa and their anti-Phytophthora activity. Antonie Van Leeuwenhoek.

[205] Gorai PS, Ghosh R, Mandal S, Ghosh S, Chatterjee S, Gond SK, Mandal NC (2021) *Bacillus siamensis* CNE6-a multifaceted plant growth promoting endophyte of *Cicer arietinum* L. having broad spectrum antifungal activities and host colonizing potential. Microbiol Res 252:126859 10.1016/j.micres.2021.126859 34536676

[206] Taechowisan T, Chanaphat S, Ruensamran W, Phutdhawong WS (2014). Antibacterial activity of new flavonoids from *Streptomyces* sp. BT01; an endophyte in *Boesenbergia rotunda* (L.) Mansf. J Appl Pharm Sci.

[207] Rajivgandhi G, Ramachandran G, Maruthupandy M, Vaseeharan B, Manoharan N (2019). Molecular identification and structural characterization of marine endophytic actinomycetes *Nocardiopsis* sp. GRG 2 (KT 235641) and its antibacterial efficacy against isolated ESBL producing bacteria. Microb Pathog.

[208] Peng C, An D, Ding W-X, Zhu Y-X, Ye L, Li J (2020). Fungichromin production by *Streptomyces* sp. WP-1, an endophyte from Pinus dabeshanensis, and its antifungal activity against *Fusarium oxysporum*. Appl Microbiol Biotechnol.

[209] Sun L, Lu Z, Bie X, Lu F, Yang S (2006). Isolation and characterization of a co-producer of fengycins and surfactins, endophytic *Bacillus amyloliquefaciens* ES-2, from *Scutellaria baicalensis* Georgi. World J Microbiol Biotechnol.

[210] Dowarah B, Agarwal H, Krishnatreya DB, Sharma PL, Kalita N, Agarwala N (2021) Evaluation of seed associated endophytic bacteria from tolerant chilli cv. Firingi Jolokia for their biocontrol potential against bacterial wilt disease. Microbiol Res 248:126751 10.1016/j.micres.2021.126751 33839507

[211] Liang Y, Wei G, Ning K, Zhang G, Liu Y, Dong L, Chen S (2021). Contents of lobetyolin, syringin, and atractylolide III in *Codonopsis pilosula* are related to dynamic changes of endophytes under drought stress. Chin Med.

[212] Pachaiyappan A, Sadhasivam G, Kumar M, Muthuvel A (2021). Biomedical potential of Astaxanthin from novel endophytic pigment producing bacteria *Pontibacter korlensis* AG6. Waste Biomass Valorization.

[213] Spina R, Saliba S, Dupire F, Ptak A, Hehn A, Piutti S, Poinsignon S, Leclerc S, Bouguet-Bonnet S, Laurain-Mattar D (2021). Molecular identification of endophytic bacteria in *Leucojum aestivum in vitro* culture, NMR-based metabolomics study and LC-MS analysis leading to potential Amaryllidaceae alkaloid production. Int J Mol Sci.

[214] Liu T-h, Zhang X-m, Tian S-z, Chen L-g, Yuan J-l (2020) Bioinformatics analysis of endophytic bacteria related to berberine in the Chinese medicinal plant Coptisteeta Wall. 3 Biotech 10:1–12 10.1007/s13205-020-2084-y PMC7002695 32099737

[215] Abdelshafy Mohamad OA, Ma J-B, Liu Y-H, Zhang D, Hua S, Bhute S, Hedlund BP, Li W-J, Li L (2020). Beneficial endophytic bacterial populations associated with medicinal plant *Thymus vulgaris* alleviate salt stress and confer resistance to *Fusarium oxysporum*. Front Plant Sci.

