# Correction to: Bioprospecting and Challenges of Plant Microbiome Research for Sustainable Agriculture, a Review on Soybean Endophytic Bacteria

**DOI:** 10.1007/s00248-022-02141-2

**Published:** 2022-11-09

**Authors:** Modupe Stella Ayilara, Bartholomew Saanu Adeleke, Olubukola Oluranti Babalola

**Affiliations:** 1 Food Security and Safety Focus Area, Faculty of Natural and Agricultural Sciences, North-West University, Private Bag X2046, Mmabatho, 2735 South Africa; 2 Department of Biological Sciences, Microbiology Unit, Faculty of Science, Olusegun Agagu University of Science and Technology, PMB 353, Okitipupa, Nigeria

**Correction to: Microbial Ecology**

**https://doi.org/10.1007/s00248-022-02136-z**

The article author (Bartholomew Saanu Adeleke) affiliations asterisks are incorrect. It should have been stated as shown above.

